# Discovery of a dual inhibitor of NQO1 and GSTP1 for treating glioblastoma

**DOI:** 10.1186/s13045-020-00979-y

**Published:** 2020-10-21

**Authors:** Kecheng Lei, Xiaoxia Gu, Alvaro G. Alvarado, Yuhong Du, Shilin Luo, Eun Hee Ahn, Seong Su Kang, Bing Ji, Xia Liu, Hui Mao, Haian Fu, Harley I. Kornblum, Lingjing Jin, Hua Li, Keqiang Ye

**Affiliations:** 1 Department of Pathology and Laboratory Medicine, Emory University School of Medicine, Atlanta, GA USA; 2 Neurotoxin Research Center of Key Laboratory of Spine and Spinal Cord Injury Repair and Regeneration of Ministry of Education, Neurological Department of Tongji Hospital, Tongji University School of Medicine, Shanghai, 200065 People’s Republic of China; 3 School of Pharmacy, Tongji Medical College, Huazhong University of Science and Technology, Wuhan, 430030 People’s Republic of China; 4 Intellectual and Developmental Disabilities Research Center, David Geffen School of Medicine at UCLA, Los Angeles, CA 90095 USA; 5 Department of Pharmacology and Chemical Biology, Emory Chemical Biology Discovery Center, Atlanta, USA; 6 Department of Radiology and Imaging Sciences, Emory University School of Medicine, Atlanta, GA 30322 USA; 7 Wuya College of Innovation, Shenyang Pharmaceutical University, Shenyang, People’s Republic of China

**Keywords:** Oxidative stress, NQO1, GSTP1, GBM, Small molecular inhibitor

## Abstract

**Background:**

Glioblastoma (GBM) is a universally lethal tumor with frequently overexpressed or mutated epidermal growth factor receptor (EGFR). NADPH quinone oxidoreductase 1 (NQO1) and glutathione-S-transferase Pi 1 (GSTP1) are commonly upregulated in GBM. NQO1 and GSTP1 decrease the formation of reactive oxygen species (ROS), which mediates the oxidative stress and promotes GBM cell proliferation.

**Methods:**

High-throughput screen was used for agents selectively active against GBM cells with EGFRvIII mutations. Co-crystal structures were revealed molecular details of target recognition. Pharmacological and gene knockdown/overexpression approaches were used to investigate the oxidative stress in vitro and in vivo.

**Results:**

We identified a small molecular inhibitor, “MNPC,” that binds to both NQO1 and GSTP1 with high affinity and selectivity. MNPC inhibits NQO1 and GSTP1 enzymes and induces apoptosis in GBM, specifically inhibiting the growth of cell lines and primary GBM bearing the EGFRvIII mutation. Co-crystal structures between MNPC and NQO1, and molecular docking of MNPC with GSTP1 reveal that it binds the active sites and acts as a potent dual inhibitor. Inactivation of both NQO1 and GSTP1 with siRNA or MNPC results in imbalanced redox homeostasis, leading to apoptosis and mitigated cancer proliferation in vitro and in vivo.

**Conclusions:**

Thus, MNPC, a dual inhibitor for both NQO1 and GSTP1, provides a novel lead compound for treating GBM via the exploitation of specific vulnerabilities created by mutant EGFR.

## Introduction

Glioblastoma (GBM) is one of the most aggressive and malignant human brain tumors with a mean survival rate of 12 months [1] despite the standard therapeutic regiment of maximal surgical resection, radiation, and chemotherapy [2]. Because GBM is highly heterogeneous, specific therapeutic targeting of GBM subclasses remains a goal in neuro-oncology. One of the major drivers of a subgroup of GBM is the epidermal growth factor receptor (EGFR). As a receptor tyrosine kinase (RTK), EGFR is implicated in cell growth and proliferation through downstream effectors such as Ras and PI-3 kinase (PI3K) and is modulated by tumor-suppressor genes NF1 and PTEN. One of the most selective genetic alterations in GBM is the amplification of EGFR, which occurs in approximately 40% of GBMs. Either wild-type or mutated forms of EGFR can be amplified. The most common mutated form lacks exons 2–7, resulting in constitutive tyrosine kinase activity (EGFRvIII) [3]. Although inhibition of EGFR activation is a tempting target, clinical trials have not proven the efficacy of this strategy. In particular, patients with EGFRvIII mutations and mutated PTEN are highly resistant to direct inhibition of the EGFR tyrosine kinase [4].

One strategy for patient-specific therapeutics is to take advantage of the downstream consequences of the activation of oncogenic mutations. Mounting evidence suggests that EGFRvIII activation specifically correlates to the level of cellular oxidative stress [5, 6]. In GBM cell lines, measurements of the intracellular and extracellular proteins indicate elevated oxidative stress specifically in the EGFRvIII-expressing cell line [7]. Subsequent studies revealed that EGFRvIII overexpression in glioblastoma cells caused increased levels of reactive oxygen species (ROS), DNA strand break accumulation and genome instability [8]. Low levels of ROS participate in cell progression and promote cell proliferation, whereas high levels of ROS induce oxidative stress and cell damage [9, 10]. Therefore, maintaining ROS homeostasis is crucial for cell growth and survival [11]. Cellular oxidative stress is generated by the imbalance of the redox status of the cell. ROS are derived from enzymatic reactions involving NADPH-dependent oxidases NAD(P)H: quinone oxidoreductase 1 (NQO1), which is a cytosolic reductase, and it plays important roles in the cellular response to numerous stresses and is upregulated in many human cancers compared to adjacent normal tissues [12]. The upregulation of NQO1 protects cells against oxidative stress by catalyzing the detoxification and the reduction of quinine substrates [13]. A recent study reports that ES936, an NQO1 inhibitor, enhances TRAIL-induced apoptosis in endometrial carcinoma Ishikawa cells [14], and inhibition of NQO1 activity leads to the effect of SPL-A on TRAIL-induced apoptosis [15].

Glutathione S-transferases (GSTs) catalyze reactions between glutathione and lipophilic compounds with electrophilic centers, leading to the neutralization of toxic compounds, xenobiotics and products of oxidative stress. It has been reported that serine phosphorylation of GSTP1 by PKCα enhances GSTP1-dependent cisplatin metabolism and resistance in human glioma cells [16]. Moreover, GST polymorphisms represent the risk factor for various cancers. For instance, GSTP1 gene Ile105Val polymorphism is involved in the development of glioma and prostate cancer and other cancers [17, 18]. Further, GSTP1 dimerizes into larger aggregates, precludes binding to JNK and inhibits its activation under ROS overexpression condition [19]. Notably, a GSTP1 inhibitor, Ezatiostat, has passed phase-II clinical trials for treating myelodysplastic syndrome, indicating that GSTP1 inhibitors might be used for human cancers [20]. Since NQO1 and GSTP1 are phase-II detoxification enzymes that reduce quinones directly to hydroquinones, eliminating the formation of ROS produced by redox cycling [21], the combination of inhibition of NQO1 and GSTP1 may offer a potential solution for cancer therapy.

In this study, we found that both NQO1 and GSTP1 were overexpressed in GBM and functioned to inhibited oxidative stress and prevent cancer cell death. Using a strategy based on high-throughput chemical screening (HTS) and affinity chromatography, we identified a small molecule NQO1 and GSTP1 dual inhibitor, MNPC, that suppressed the proliferation and stimulated apoptosis in a highly passaged cell line and primary GBM cells bearing the EGFRvIII mutation. The co-crystal structure between MNPC and NQO1, and the molecular docking of MNPC with GSTP1 revealed that MNPC blocked the active sites in both enzymes. MNPC also blocked GBM propagation and prolonged the survival rate in mice-bearing orthotopic tumors derived from EGFRvIII-positive cells. Thus, our findings demonstrate that a small molecule dual inhibitor for both NQO1 and GSTP1, downstream of EGFRvIII, provides a novel strategy for GBM therapy by disrupting the redox homeostasis.

## Materials and methods

### Cell lines and cell culture

The human glioblastoma cell line U87MG was stably transfected with vector control, pLHCX-EGFRvIII, pGFP-NQO1 and pRS-GSTP1, which were supplemented with various antibiotics. For EGFRvIII, 150 μg/mL of hygromycin was used; for pGFP-NQO1 and pRS-GSTP1, 0.7 μg/mL of puromycin was employed. The cells were supplemented with 10% fetal bovine serum (Hyclone, USA), penicillin (100 U/ml) and streptomycin (100 U/ml) (ABAM Life Technologies, USA) in a humidified incubator with 5% CO_2_ at 37 °C.

### Ultra-high-throughput screening (uHTS) technology

The development of the uHTS enabled the discovery of genomic selective cancer cell growth inhibitors. The uHTS CellTiter Blue cell viability assay in 1536-well format rapidly screens for the compounds that selectively kill GBM cells with EGFRvIII cells. A parallel assay system was established for this purpose. The uHTS campaign was carried out at the Emory Chemical Biology Discovery Center (ECBDC) [22]. Screening data were analyzed using CambridgeSoft Bioassay software [23]. Z’ factors are greater than 0.5 across the screening plates, indicating a robust assay for uHTS. Z’ factor is calculated with the following equation: Z’ = 1-(3SD background + 3SD control)/(FI control – FI background). The background is defined by the average fluorescence intensity (FI) signal from wells with medium and without cells. The DMSO control is defined by the FI signal from wells with cells and with 1% vehicle (DMSO), but without compound. 0.5 < Z’ < 1 indicates a robust assay for HTS [24]. The effect of the compound on the cell growth and proliferation was expressed as % of control based on per plate and is calculated as the following equations:

$$\% \;{\text{of}}\;{\text{Control}} = \left( {{\text{FI}}\;{\text{compound}}{-}{\text{FI}}\;{\text{blank}}} \right)/\left( {{\text{FI}}\;{\text{DMSO}}\;{\text{control}}{-}{\text{FI}}\;{\text{blank}}} \right) \times {1}00.$$

### In vitro functional analysis: sphere formation

For sphere formation experiments, cell numbers were calculated and cells were plated into 96-well plates at a density of 100, 50, 25, and 12 cells per well (24 wells for each density). Cells were kept in the incubator for 2 weeks before sphere formation was assessed and images were taken. Spheres larger than 10 cells in diameter were considered for analysis. The number of wells forming spheres was used as input to the Walter and Eliza Hall Institute Bioinformatics Division ELDA analyzer (https://bioinf.wehi.edu.au/software/elda/) in order to obtain stem cell frequencies [25].

### Affinity chromatography using epoxy-activated agarose

In order to produce alcohol or TIZ-agarose, 0.75 g lyophilized epoxy-activated agarose with a C_12_ spacer was suspended in 10 ml distilled water and centrifuged at 500 rpm for 8 min. Washing in water was repeated twice, with one of which using the coupling buffer (0.1 M NaHCO3, pH 9.5). After the last wash, 15 mg TIZ or 200 μl alcohol was added and the coupling buffer was added to a maximum volume of 5 ml. The mixture was incubated overnight at 37 °C under slow but continuous shaking in order to allow the coupling of the epoxy group to TIZ or alcohol. The resulting column medium was then transferred to a chromatography column (Novagen, Merck, Darmstadt, Germany), and the column was washed with coupling buffer (20 ml). After that, it was washed with ethanolamine (1 M, pH 9.5) for room temperature overnight in order to block residual reactive groups. Finally, the column was extensively washed with PBS and PBS-DMSO (1:1) to remove unbound TIZ or alcohol. Protein extraction from U87MG/EGFRvIII cells was loaded with a flow rate of 0.1 ml/min. The column was washed with PBS until the baseline was flat. Proteins binding to alcohol or TIZ columns were incubated with 1 mM MNPC overnight. PBS followed by elution with a pH shift (100 mM glycine, pH 2.9) in order to remove nonspecifically bound proteins [26]. Silver staining was applied according to the method of the previous study [27].

### Mass spectrometry analysis

The protein samples were in-gel digested with 10 ng/μl Glu-C. Then, the peptide samples were resuspended in loading buffer (0.03% trifluoroacetic acid, 0.1% formic acid, and 1% acetonitrile), then loaded onto a 20-cm nano-HPLC column and finally generated by a Dionex RSLCnano UPLC system (Thermo, USA). Peptides were ionized with 2.0 kV electrospray ionization voltage from a nano-ESI source on an Orbitrap Fusion mass spectrometer (Thermo, USA) [28].

### Binding affinity analysis using BIAcore surface plasmon resonance

Experiments were performed on a Biacore X100 system (Biacore AB, Uppsala, Sweden). Recombinant human NQO1 and GSTP1 dissolved in 10 mM sodium acetate buffer (pH = 5.0) were covalently immobilized in the dextran matrix of a CM5 sensor chip with the Amine Coupling Kit using a standard primary amine coupling procedure. The compound MNPC was injected into the flow cells in running buffer at a flow rate of 30 μL/min for 120 s of association phase, followed by a 120-s dissociation phase and a 30-s regeneration phase. The surface of the sensor chip was regenerated via the injection of 10 μL of the regeneration buffer (5 mM NaOH). The association rate constant k_a_ and dissociation rate constant k_d_ were calculated and analyzed using the monovalent analyte model, and the equilibrium dissociation constant (KD) was calculated (KD = k_d_/k_a_) [29].

### GSTP1 Kinetic assay

The enzymatic activity of GSTP1 and its mutations was measured through the increased absorbance at 340 nm, which derived from the conjugation of reduced glutathione (GSH) and 1-chloro-2,4-dinitrobenzene (CDNB). The conjugation was initiated by adding CDNB to the mixture of GSH and GSTP1 or its mutants, and OD was immediately and continually measured for 20 min at 25 °C. The final assay mixture included 1 mM CDNB, 1 mM GSH in the buffer of 100 mM potassium phosphate pH 6.5, 1 mM EDTA. The inhibitory potency of MNPC versus GSTP1 or mutants was determined by calculating the residual activity after incubated with MNPC for 30 min at room temperature. Parallel control was run to monitor spontaneous conjugation of GSH and CDNB in the absence of the enzyme, and absorbance change of the control was used for the correction of an enzymatic reaction. The inhibition rate in triplicate was calculated by GraphPad Prism software (GraphPad, San Diego, CA) to give IC_50_ using nonlinear regression analysis.

### Microscale thermophoresis (MST)

Since Tris buffer is incompatible with MST labeling, GSTP1 and its mutants were performed an initial buffer exchange to 20 mM HEPES pH 7.6, 200 mM NaCl before labeling. Then, the proteins were fluorescently labeled using Monolith Protein Labeling Kit RED-NHS 2^nd^ generation dye (NanoTemper Technology, Munich, Germany) according to the protocol. MNPC was prepared with a series of concentrations (varying from 2 mM to 11 nM) at a dilution ratio of 1: 2. Six microliters of the fluorescence-labeled GSTP1 or mutations was mixed with 6 μl of variable concentration of MNPC and incubated for 10 min at room temperature. The mixtures were loaded into standard glass capillaries and analyzed on Monolith NT.115 at 25 °C, with 40% LED power and 100% Laser power in triplicate. Data were analyzed by NTAnalysis software to calculate the K_d_ values.

### Protein expression and purification

Human NQO1 (GenBank: NP_000894.1) and GSTP1 (GenBank: AAH10915.1) genes were cloned into pET28a with an N-terminal hexahistidine tag separated by a thrombin site and expressed in *Escherichia coli* strain BL21 (DE3). Bacterial culture was grown in LB medium with 35 μg/ml of kanamycin at 37 °C until OD_600_ reached 0.6 to 0.8 and then induced by adding 0.4 mM isopropyl-L-thio-B-D-galactopyranoside (IPTG) for 16 h at 20 °C. Recombinant NQO1 proteins were purified as follows: after harvest by centrifugation, cells were lysed in 10% glycerol, 1% TritonX-100, 200 mM NaCl, 10 mM imidazole and 100 mM Tris (pH 7.6) supplemented with 1 mM phenylmethanesulfonyl fluoride (PMSF). Soluble protein was separated from the cleared cell lysate by centrifugation at 21,000 g 40 min, then submitted to Ni–NTA resin (Qiagen) with an elution buffer of 200 mM NaCl, 150 mM imidazole and 20 mM Tris (pH 7.6). Protein was then concentrated and loaded onto a Superdex 200 10/300 GL (GE Healthcare) pre-equilibrated with 200 mM NaCl, 20 mM Tris (pH 7.6). Recombinant GSTP1 protein was purified as described above, except with a slight difference in buffer composition. For GSTP1, β-mercaptoethanol was added to all buffers to a final concentration of 2 mM. The purity of NQO1 and GSTP1 was confirmed by SDS-PAGE and Coomassie blue staining.

### Crystallization and structure determination

Crystals of the NQO1 complex with MNPC were obtained by co-crystallization with the sitting drop vapor diffusion method. Purified NQO1 was concentrated to 12 mg/mL and then incubated with MNPC at a molar ratio of 1:3 over ice for 1 h. One microliter of NQO1-MNPC solution was mixed with 1 μL of mother liquor and further equilibrated with reservoir solution at 20 °C. Crystals appeared in a week, with a crystallization condition of 0.2 M lithium sulfate, 1.8 M ammonium sulfate, 0.1 M imidazole pH 7.0. The crystals were cryoprotected using the crystallization solution with 20% glycerol and then flash-frozen directly into liquid nitrogen.

The attempt was also made to obtain crystals of the GSTP1–MNPC complex. GSTP1 with a concentration of 10 mg/ml was used for crystallization, and the solution of the GSTP1–MNPC mixture was generated just as NQO1-MNPC. Crystals appeared in one day or two in the condition of 0.1 M MES PH5.4, 30% PEG8000, 10 mM DTT, 20 mM CaCl_2_, and grew in a week to the maximum size at 20 °C. After crystals grew to the full size, the crystallization condition was supplemented with MNPC of final concentration 3 mM. After soaked for 4 h, crystals were then flash-frozen in liquid nitrogen until data collection.

Diffraction data were collected at the Shanghai Synchrotron Radiation Facility (SSRF) at beamline 17U1, 18U1 and 19U1. The data were measured from a single crystal maintained at 100 K at a wavelength of 0.9789 Å, and the reflections were indexed, integrated, and scaled by HKL2000 [30]. The structure of NQO1–MNPC complex was solved by molecular replacement using the program PHASER in the PHENIX package [31] with the search model of PDB ID 2F1O for NQO1, 3GUS [32] for GSTP1, followed by repeated cycles of model building with　Coot [33] and refinement with REFMAC [34] and PHENIX, yielding the published NQO1–MNPC complex structure (PDB ID 6LLC). The solvent, ligand and inhibitor were built into the density in later rounds of the refinement. Data collection and refinement statistics are shown in Extended Table 2.

### Molecular docking

ICM 3.8.2 modeling software on an Intel i7 4960 processor (MolSoft LLC, San Diego, CA) was used to perform molecular docking. GSTP1 model was obtained for protein data bank (PDB ID 3GUS) (Federici et al., 2009). MNPC was input as the 3D compound and calculated according to the internal coordinate mechanics (Internal Coordinate Mechanics, ICM) [35].

### Cell proliferation assays

In vitro assessment was implemented for cell proliferation using the 3-(4, 5- dimethylthiazol- 2-yl)-2,5-diphenyltetrazolium bromide (MTT) assay. After different treatments with specific time periods, 10 μL of MTT (5 mg/mL in PBS, Sigma, USA) was added to each well and the plates were incubated for 3 h at 37 °C. The resulting formazan product was dissolved with DMSO. The absorbance at a wavelength of 490 nm was recorded using a microplate reader (BioTek Instruments Inc., USA).

### ROS measurement

The DCFH-DA method was employed to detect the levels of intracellular reactive oxygen species (ROS). After various treatments, the cells were collected and then incubated with 5 μM DCFH-DA (ROS dye, #C6827, Invitrogen, USA) for 1 h at 37 °C. The fluorescence intensity was measured by the microplate reader (BioTek Instruments Inc., USA) with settings at excitation and emission equal to 485/535 nm.

### LDH, protein carbonyl assay and GSH/GSSG ratio measurements

After collecting the supernatants, the cytokine concentrations were measured using LDH assay kits (Promega Corporation, USA). Protein carbonyl level and GSH/GSSG ratio were measured from cell homogenates using Protein Carbonyl Assay Kit (#ab126287, Abcam, Cambridge MA, USA) and GSH/GSSG-Glo™ (Promega Corporation, USA), respectively.

### Protein extraction and western blot analysis

After different treatments with the conditions described, the cells were harvested and the total proteins were extracted. Equal amounts of the proteins were loaded on SDS-PAGE and western blot assays were analyzed. Primary antibodies were from CST, USA, and the following targets were used: p-EGFR (#2236); EGFR (#2232); NQO1 (#3187); GSTP1 (#3369); cleaved caspase 3 (#9664); β-actin (#3700).

### AEP activity assay

After different treatments, cell lysates were incubated in 200 μl assay buffer (20 mM citric acid, 60 mM Na_2_HPO_4_, 1 mM EDTA, 0.1% CHAPS and 1 mM DTT, pH 6.0) containing 20 μM AEP substrate Z-Ala-Ala-Asn-AMC (Bachem, USA). AMC released by substrate cleavage was quantified by measuring every 10 min at 460 nm in a fluorescence plate reader at 37 °C in kinetic mode for the total time of 1 h.

### Migration assays

A total of 1 × 10^4^ cells were seeded onto the upper part of a transwell chamber (BD Bioscience, USA) containing a membrane filter for migration assays. Serum-free medium was added to the upper well, and medium containing 10% FBS was added to the lower well. After different treatments and incubation at 37 °C with 5% CO_2_, the filters were stained with crystal violet. Five random fields were counted per chamber by using a microscope.

### NQO1 activity assays

NQO1 activity was measured essentially as described previously. Assays were performed in 25 mM Tris–HCl, pH 7.4, 0.7 mg/mL BSA, 250 mM sucrose, 0.2 mM NADPH and 40 μM dichlorophenolindophenol (DCPIP) in the presence of NQO1 protein with 10, 5, 2.5, 1.25, 0.625, 0.3125, 0.15625 and 0 μM of MNPC. It is analyzed for the reduction of DCPIP measured at 600 nm [36].

### TUNEL assay

After different treatments, the cells and the tumor tissues were processed with Apo-Direct TUNEL Assay (Roche Applied Science, Germany) following the manufacture’s instruction. The slides were photographed with a fluorescence microscope (Nikon, Japan).

### Transfection and infection of the cells

The NQO1-siRNA(#sc-37139), GSTP1-siRNA (#sc-72091) and nontargeting siRNA (#sc-37007) as control were purchased from Santa Cruz Biotechnology. HA-NQO1 plasmid was purchased from Addgene. Myc-DDK-GSTP1 (#RC203086) was purchased from Origene. The U87 MG and U87MG/EGFRvIII cells were transfected with 20 nM siRNA or 2 μg plasmid using the Lipofectamine 3000 and P3000 (#L3000075, Invitrogen, USA) according to the manufacture’s protocol.

### In vivo mouse model experiments

Animals were housed, maintained and treated at Emory University in accordance with protocols approved by the Institutional Animal Care and Use Committee (IACUC) at Emory University. For xenograft animal models, U87MG/EGFRvIII cells (2 × 10^6^) in 100 μl of PBS were inoculated subcutaneously into 6-week-old nude mice. Tumor growth was assessed every 3 days via size and body weight measurements. The total tumor volume (TV) was calculated according to the following formula: TV (mm^3^) = a * b^2^/2, where “a” is the minimum diameter and “b” denotes the maximum diameter. The mice were euthanized after 28 days. For the intracranial model, mice were placed in a stereotaxic instrument, and cells injection (1 × 10^5^) in 2 μl was performed stereotaxically at coordinates anteroposterior (AP) -2.0 mm and mediolateral (ML) + 0.7 mm relative to bregma, and dorsoventral (DV) -3.0 mm from the dural surface. The needle remained in place for 5 min before it was removed slowly. The mice were placed on a heating pad until it began to recover from the surgery. After the surgery for 8 days, intraperitoneal (i.p.) injection was used at dose levels of 3 mg/kg, 10 mg/kg or control agent. This treatment was performed every 2 days for a total of 10 times. After the drug treatment, mice were euthanized and the tumor volumes were analyzed by MRI [37].

### Hematoxylin–eosin (H&E) staining and immunohistochemistry

The tumors and primary organs from the nude mice of the above models were fixed in 10% formalin overnight and were then embedded in paraffin. Sections were prepared, and H&E staining was conducted to detect any histological changes of the tumors and organs. The expression of Ki67 in the tumor tissue slices was assessed using a technique that has been reported previously. Photographs were taken using a fluorescence microscope (Nikon, Japan).

### Bioinformatic analysis

Bioinformatic data analysis was obtained from the TCGA data portal (https://cancergenome.nih.gov/dataportal/data/about), UALCAN (https://ualcan.path.uab.edu/index.html) [38], GlioVis (https://gliovis.bioinfo.cnio.es)[39], respectively.

### Statistical analysis

Data visualization and analysis were performed with GraphPad Prism 6 (GraphPad Software Inc., La Jolla, CA, USA). Statistical analysis was performed using either Student’s t test or one-way ANOVA. Significant difference among groups was assessed as *p < 0.05; **p < 0.01; ***p < 0.001.

## Results

### High-throughput screening for small molecules that selectively inhibit proliferation of U87MG/EGFRvIII cells versus U87MG cells

**Fig. 1:**
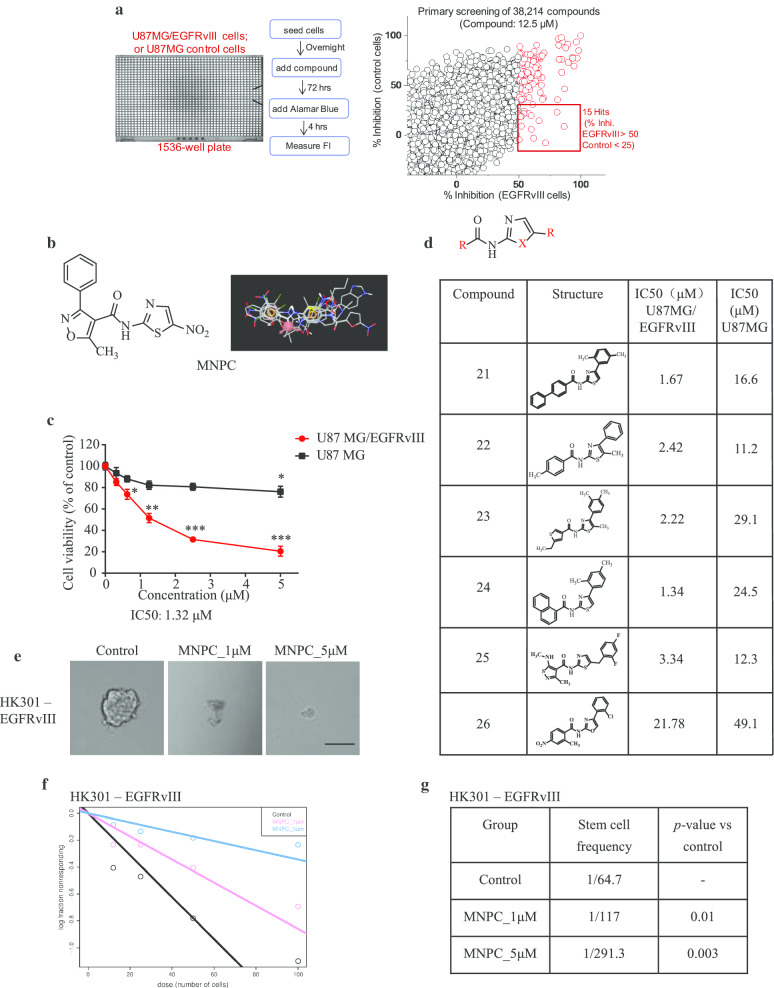
Identification of MNPC via ultra-high-throughput screen (uHTS) assay for U87MG/EGFRvIII versus U87MG cells. **a** Miniaturized uHTS cell viability assay development for selectively blocking U87MG/EGFRvIII versus U87MG cells. **b** The chemical structure and 3D alignment of the structure of MNPC. **c** Effect of MNPC (0, 0.3125, 0.625, 1.25, 2.5, 5 μM for 72 h) on the proliferation of U87MG/EGFRvIII and U87MG cells. Data are mean ± SD from three replicates, *p* values were determined by a two-tailed Student’s t test (*p < 0.05; **p < 0.01; ***p < 0.001). **d** The SAR analysis of MNPC. The structures and IC50 of inhibition activity of the compound derivatives in U87MG/EGFRvIII and U87MG cells. **e**–**g** MNPC dose-dependently blocks primary GBM sphere formation capacity. Primary GBM cells with EGFRvIII mutations were cultured with two doses of MNPC and plated for limiting dilution analysis. The formation of spheres (shown in **e**) was assessed for all wells and used to generate stem cell frequencies (**f**, **g**). Scale bar: 100 μm

To search for the pharmacological agents for potential GBM therapeutics, we employed an ultra-high-throughput screen (uHTS) in a 1536-well plate format and testing funnels to screen and identify the small molecules, which differentially inhibited the growth of U87MG/EGFRvIII cells against U87MG (PTEN mutant) parental cells. While the precise origin of U87MG cells is in the debate, recent studies demonstrate that it is GBM-derived and thus appropriate for these screening studies [40]. After screening more than 38,000 compounds, we obtained 15 positive hits with different chemical backbones that showed > 50% inhibition of cell viability in U87MG/EGFRvIII cells but < 25% inhibition in U87MG cells (Fig. 1a). After the structure–activity relationship (SAR) analysis, we found that most compounds contained two aromatic rings bridged through an amide group. Namely, these compounds possessed N-phenylbenzamide or N-phenylthiophene/furan-2-carboxamide moieties. The representative compound MNPC (5-methyl-N-(5-nitro-thiazol-2-yl)-3-phenylisoxazole -4-carboxamide) displayed an IC_50_ of 1.32 μM in inhibiting U87MG/EGFRvIII cell proliferation (Fig. 1b and c). SAR studies also demonstrated that 3-phenyl-5-methyl isoxazole group and the nitro group on MNPC could be replaced without substantially affecting the inhibitory activity (Additional file 1: Supplementary Fig. 1), suggesting that these groups are not essential for the inhibitory function. On the other hand, compared to oxazole, thiazole substitution increased the inhibitory effect (Fig. 1d), indicating that a size increase in X position elevates its binding to the target. Hence, the MNPC was selected for further study for its anticancer functions. To determine whether MNPC exerts similar effects on primary GBM cells, we performed limiting dilution sphere-forming assays on cultures with EGFRvIII mutation as well as two other EGFR wild-type cultures. MNPC diminished sphere formation and dramatically suppressed the growth of spheres in the EGFRvIII mutant cultures (Fig. 1e-g and Additional file 1: Supplementary Fig. 2a) with little or no effect on the EGFR wild-type cultures (Additional file 1: Supplementary Fig. 2b and c). Hence, MNPC selectively blocks the proliferation of a standard cell line and primary GBM cells bearing the EGFRvIII mutation.

**Fig. 2:**
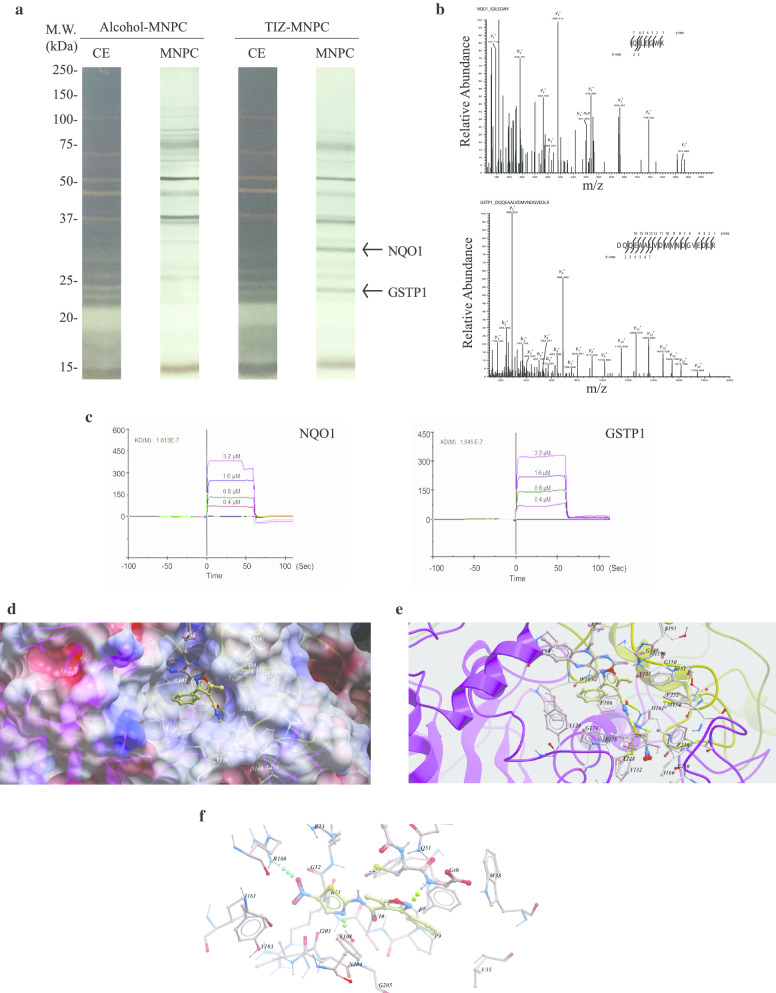
NQO1 (NAD(P)H dehydrogenase quinone 1) and GSTP1 (glutathione S-transferase Pi 1) are the major MNPC-binding proteins. **a** Affinity chromatography of cell-free extract from U87MG/EGFRvIII cells. Aliquots of fractions were separated by SDS-PAGE. Bands were visualized by silver staining. CE, crude extract. MNPC, protein eluted with 1 mM MNPC in PBS **b** Amino acid sequence of the 29 kDa and 25 kDa bands that were identified by MS/MS as NQO1 and GSTP1. **c** BIACore X-100 binding assay for NQO1 and GSTP1. The kinetic parameters KD were measured by Biacore evaluation software. **d** X-ray crystal structure of NQO1 in complex with MNPC (PDB ID 6LLC). Surface representation of NQO1 in complex with MNPC bound in the active pocket formed in the dimer interface. The benzene ring of MNPC (yellow) was sandwiched by Phe178 and FAD (Brown) by π–π stacking. **e** Key interactions between MNPC and NQO1, including hydrogen bond with His161 and hydrophobic interactions with Tyr126, Tyr128, M131, Phe236 from one monomer and Trp105, Phe106, Met154, Tyr155 from another monomer. **f** Interactions between MNPC and GSTP1 are generated by molecular docking. Key interactions between MNPC and GSTP1, including hydrogen bonds with Arg100, Tyr108 and GSH, π–π stacking with Phe8 and Tyr108

### NQO1 and GSTP1 are the major MNPC-binding targets

Since MNPC was the most active compound against EGFRvIII-expressing GBM in the cell growth inhibition experiments, we sought to identify the cellular targets of this compound. Further SAR study revealed that nitazoxanide (NTZ) and tizoxanide (TIZ), two FDA-approved drugs for treating antiparasitic and cryptosporidium infection, displayed anti-proliferative activities in U87MG/EGFRvIII cells to a comparable extent as MNPC (Additional file 1: Supplementary Fig. 2d and e). This finding supported the hypothesis that 5-nitro-thiazol-4-carboxamide is the crucial pharmacophore for MNPC to exert its selective anticancer activity. To determine candidate protein targets that bind both TIZ and MNPC, we subjected U87MG/EGFRvIII cytoplasmic extracts to an affinity column prepared by covalently coupling TIZ to the epoxy-agarose via its phenol group with subsequent elution with free MNPC solution. Two major proteins with the molecular weight of 24 kDa and 29 kDa, as visualized by silver staining, were specifically observed in the MNPC fraction but not in the control fraction. Proteomic analysis of the eluted proteins revealed their identities: NQO1 and GSTP1 (Fig. 2a and b). To determine whether MNPC, indeed, specifically binds to NQO1 and GSTP1, we performed the BIACORE binding assay by immobilizing MNPC to the chip and found that MNPC exhibited high affinity to NQO1 and GSTP1 recombinant proteins, respectively (Fig. 2c).

Next, we obtained the co-crystals of MNPC with NQO1 (PDB ID 6LLC) and GSTP1 (PDB ID 6LLX). The crystal structure of MNPC in complex with NQO1 was solved at 2.5 Å (Fig. 2d and e) and revealed that MNPC bound with NQO1 at the same active site as the known inhibitor dicoumarol (PDB ID 2F1O) [41], where MNPC deeply resided in the pocket and was oriented above the isoalloxazine ring of bound cofactor FAD by π-π stacking interaction. The active pocket of NQO1 was formed by two enzyme monomers with head and tail inlaid to each other, in which several aromatic amino acids composed a relatively hydrophobic pocket to contain FAD and MNPC. Phe178 and the isoalloxazine ring of FAD formed π-π stacking sandwich with the benzene ring of MNPC. Tyr126, Tyr128, M131, Phe236 from one protein monomer and Trp105, Phe106, Met154, Tyr155 from another monomer formed strong hydrophobic interactions with MNPC. Interestingly, the hydrogen bonding between His161 with the amide carbonyl group of MNPC and the hydrophobic interaction between M131 with the thiazole ring of MNPC locked MNPC in a spatial orientation in which the plane of thiazole ring was perpendicular to the plane of the benzene ring and FAD. Attempts at co-crystallization between MNPC with GSTP1 revealed some electron density in the putative inhibitor binding site. However, it could not be fitted for the whole MNPC molecule. Therefore, we performed the molecular docking and subsequent mutagenesis analysis to elucidate the binding mode of MNPC with GSTP1. From the generated docking model, we found that MNPC was located in the active pocket of GSTP1, closely adjacent to the substrate GSH. It formed two hydrogen bindings with Arg100 and Tyr108 and one hydrogen bonding with GSH. MNPC also exhibited the π-π stacking with Phe8 and Tyr108 (Fig. 2f). To further verify the docking results, site-directed mutagenesis was conducted to generate several mutants (F8A, R100A, V104A, Y108A, F8A/Y108A), and inhibition of enzymatic activity against these mutants was measured. As expected, inhibitory activities of MNPC against mutants F8A, R100A, Y108A, F8A/Y108A were much weaker than that of wild-type GSTP1 and V104A mutant, with IC_50_ of 2.42 ± 0.36 μM, 2.37 ± 0.38 μM, 25.74 ± 1.41 μM, 4.01 ± 0.60 μM, respectively, while MNPC had an IC_50_ of 0.40 ± 0.39 μM and 0.65 ± 0.19 μM against wild-type GSTP1 and V104 mutant, respectively (Additional file 1: Supplementary Table 1, Additional file 1: Supplementary Fig. 2f and 2g). Importantly, the inhibition of MNPC decreased the most with Y108A mutant, which was consistent with the docking results that Tyr108 formed both hydrogen bonding and hydrophobic interaction with MNPC. To further verify these observations, a microscale thermophoresis (MST) assay was employed to measure the binding ability of MNPC with GSTP1 and its mutants. While K_d_ values of wild-type GSTP1 and V104A mutant were 17.60 ± 0.80 μM and 24.40 ± 1.95 μM, respectively, other mutants showed no detectable binding. Taken together, the data show that Tyr108 is the most important amino acid for binding of GSTP1 with MNPC, while Phe8 and Arg100 also participate in the interaction between MNPC and GSTP1. Val104 is not involved in the interaction and its mutant could then act as a negative control.

### MNPC inhibits both NQO1 and GSTP1 and induces the oxidative stress in U87MG/EGFRvIII cells

**Fig. 3:**
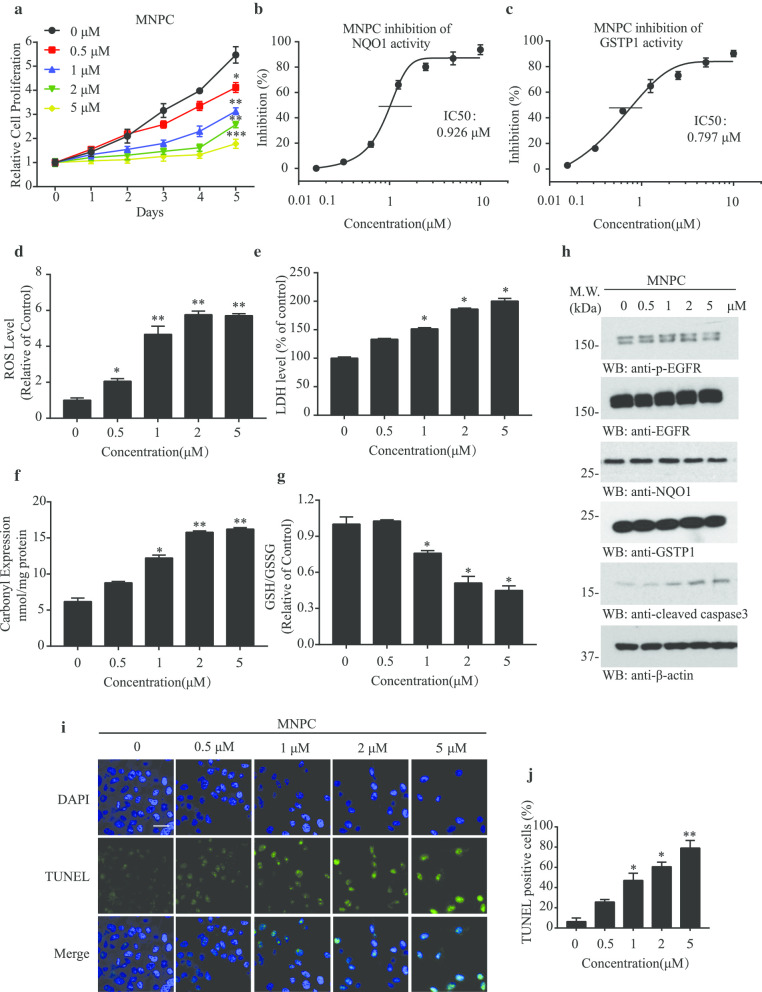
MNPC inhibits NQO1 and GSTP1 and mediates the oxidative stresses in U87MG/EGFRvIII cells. **a** MNPC inhibits U87MG/EGFRvIII cell proliferation in a dose-dependent manner. Cell proliferation of U87MG/EGFRvIII cells treated with MNPC at the dose of 0, 0.5, 1, 2 and 5 μM for 5 days, and MTT assay was conducted. **b** NQO1 and **c** GSTP1 enzyme activity inhibition by MNPC. **d** The ROS levels, **e** LDH levels, **f** Carbonyl Expression and **g** GSH/GSSG levels in U87MG/EGFRvIII cells after treatment with MNPC. **h** Western blot analysis of U87MG/EGFRvIII cells treated with different concentrations of MNPC. **i** Effect of MNPC on apoptosis confirmed by TUNEL assay. The TUNEL assay was carried out by TUNEL Assay Kit. The images of TUNEL positive cells were captured by a confocal microscope. Scale Bar: 20 μm. The cells were treated with 0, 0.5, 1, 2 and 5 μM MNPC for 48 h. **j** The bar graph represents the quantification of TUNEL assay. Data are means ± SEM (*p < 0.05; **p < 0.01; ***p < 0.001, n = 3)

To explore the anti-proliferative effects of MNPC, we treated U87MG/EGFRvIII cells with different doses of MNPC (0, 0.5, 1, 2 and 5 μM) for 5 days and found that it dose-dependently inhibited the growth of the cultures (Fig. 3a). Cell proliferation and LDH assays demonstrated MNPC barely affected the cell proliferation of normal cells (Additional file 1: Supplementary Fig. 3a and b), indicating that the toxicity is negligible. We found that MNPC had no effect on migration (Additional file 1: Supplementary Fig. 3c), nor inhibited a key enzyme AEP (asparagine endopeptidase) (Additional file 1: Supplementary Fig. 3d) in migratory processes [42]. Since NQO1 and GSTP1 were the major binding proteins of MNPC, we conducted in vitro reductase enzyme activity assays with purified recombinant proteins and found that MNPC inhibited NQO1 and GSTP1 activities with IC_50_ values of 0.926 and 0.797 μM, respectively (Fig. 3b and c). NQO1 acts as an antioxidant enzyme by regenerating antioxidant forms of ubiquinone and vitamin E quinone [43]. We also examined the cellular ROS concentrations and cytotoxicity after MNPC treatment. MNPC treatment resulted in elevated ROS levels and induced LDH release and carbonyl expression in U87MG/EGFRvIII cells, while downregulating GSH/GSSH ratios in a dose-dependent manner (Fig. 3d-g). Hence, MNPC may regulate oxidative stress and modulate redox homeostasis via inhibiting both NQO1 and GSTP1. Notably, the protein levels of NQO1 and GSTP1 were not changed upon MNPC treatment. Thus, MNPC inhibited GSTP1 and NQO1 enzyme activities without promoting a reflex upregulation of GSTP1 and NQO1 levels that could potentially mediate resistance. MNPC treatment-induced caspase 3 activation in U87MG/EGFRvIII cells (Fig. 3h), indicating that the escalated oxidative stress triggers apoptosis. The results were also confirmed by the TUNEL assay (Fig. 3i). In sum, these findings suggest that reducing oxidative stress by reductive enzymes like NQO1 and GSTP1 is critical for GBM cell proliferation.

### NQO1 and GSTP1 mediate U87MG/EGFRvIII oxidative stress and cell death

**Fig. 4:**
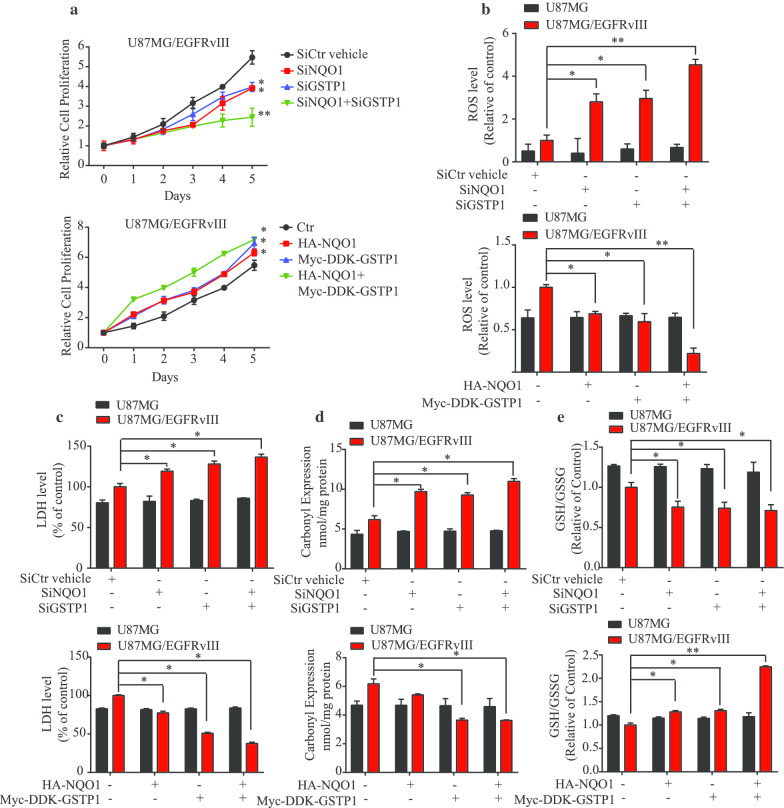
NQO1 and GSTP1 regulate cancer cell proliferation and oxidative stress. **a** Cell proliferation rates were determined by MTT assay. U87MG/EGFRvIII cells were transfected with siRNAs for depleting NQO1, GSTP1 or both. The cell proliferation was determined at different time points (upper). U87MG/EGFRvIII cells were transfected with various constructs for overexpressing NQO1, GSTP1 or both. The cell proliferation was determined at different time points (lower). The effect of knockdown and overexpression NQO1 and GSTP1 in the U87MG/EGFRvIII cells. **b** ROS levels, **c** LDH levels, **d** Carbonyl expression levels and **e** GSH/GSSG ratios. Data are means ± SEM (*p < 0.05; **p < 0.01; ***p < 0.001, n = 3)

To validate the roles of NQO1 and GSTP1 in the specific oxidative defense of EGFRvIII-expressing cells, we used knockdown and overexpression strategies. Knocking down either NQO1 or GSTP1 selectively decreased the cell proliferation in U87MG/EGFRvIII cells but not U87MG cells, and knockdown of both enzymes in combination produced an even greater effect (Fig. 4a and Additional file 1: Supplementary Fig. 4a). As expected, the ROS levels and LDH levels were preferentially increased in U87MG/EGFRvIII but not U87MG cells upon NQO1 and GSTP1 deletion (Fig. 4b and c). Protein carbonyl expression and GSH/GSSG ratios confirmed these findings (Fig. 4d and e). NQO1 and GSTP1 knockdown activated apoptotic caspase 3 in U87MG/EGFRvIII cells (Additional file 1: Supplementary Fig. 4b), fitting with MPNC’s pro-apoptotic effect. Overexpression of NQO1 and GSTP1, on the other hand, resulted in diminished ROS and enhanced cell growth in EGFRvIII-expressing but not EGFR-WT U87MG cells (Fig. 4 and Additional file 1: Supplementary Fig. 4). The studies thus far indicate that NQO1 and GSTP1 preferentially regulate the oxidative stress and cell viability in U87MG/EGFRvIII in comparison with U87MG cells.

### NQO1 and GSTP1 regulate GBM progression in vivo

**Fig. 5:**
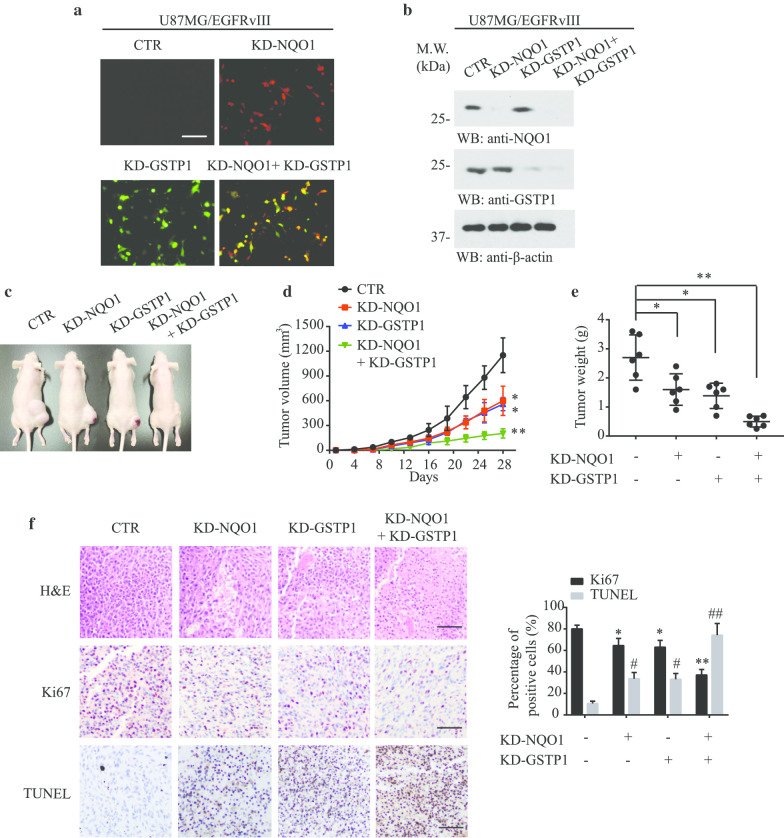
The anticancer effect of depleting NQO1 and GSTP1 in the nude mice model. **a** U87MG/EGFRvIII cells were stably knocked down with NQO1, GSTP1 or both. The images of stable cell lines were captured by a fluorescence microscope. Scale bar: 50 μm; **b** Immunoblotting validated that protein levels of GSTP1 and NQO1 in four different stable cell lines. **c** Tumor growth suppression by depletion of both NQO1 and GSTP1 in nude mice. **d** Tumor volume curve. Tumor growth suppression in nude mice by both depletion of NQO1 and GSTP1. **e** Tumor weight of the mice at the endpoint. **f** H&E, Ki67 and TUNEL staining of tumor slices in various groups. Scale bar: 100 μm. Significant differences are considered when *p < 0.05; **p < 0.01; ***p < 0.001

To further examine the effects of NQO1 and GSTP1 on U87MG/EGFRvIII cancer cell proliferation in vivo, we constructed stable knockdown cell lines that expressed their specific shRNAs. The transfection efficiency was validated by fluorescence microscopy and Western blotting, as presented in Fig. 5a and b. Next, U87MG/EGFRvIII cells stably transfected with sh-NQO1 or sh-GSTP1 or both were subcutaneously inoculated into nude mice. Representative photographs of mice from each group were taken at the endpoint (Fig. 5c). As shown in Fig. 5d, knockdown of either NQO1 or GSTP1 separately significantly decreased tumor volumes and weight, and combined knockdown resulted a much greater effect. H&E staining of tumor slices in dual knockdown groups revealed tumor tissue damage and morphology differences such as sparser cellularity as compared to other groups. Immunohistochemical (IHC) staining for Ki67 showed an almost 40% reduction in the number of proliferating cells in the dual inhibition group compared to the control group. TUNEL assay indicated extensive apoptosis in tumor tissues, suggesting that depletion of either NQO1 or GSTP1 triggers apoptosis, with the combined knockdown producing more extensive apoptotic cell death than the other groups (Fig. 5f). Thus, NQO1 and GSTP1 played a critical role in EGFR-vIII-positive GBM cell survival in vivo, supporting that they are the promising targets for GBM therapy.

### NQO1 and GSTP1 are highly positively correlated in GBM

To gain insight into the roles of NQO1 and GSTP1 in human GBM, we analyzed a dataset from The Cancer Genome Atlas (TCGA) featuring expression and the corresponding clinical data of selected GBM patient samples. Notably, NQO1 and GSTP1 expression levels were significantly increased in the samples of high grade to low grade (Additional file 1: Supplementary Fig. 5A). Consistent with our findings in primary GBM cells, a significant positive correlation was observed between the expression of NQO1 and GSTP1 (Additional file 1: Supplementary Fig. 5B). When compared with normal brain tissues, GSTP1 was highly expressed in GBM patient samples (Additional file 1: Supplementary Fig. 5C), with no significant sex difference (Additional file 1: Supplementary Fig. 5D). However, NQO1 expression only slightly changed in different ages of patients (Additional file 1: Supplementary Fig. 5E). Interestingly, both NQO1 and GSTP1 expression levels were higher in colon and lung cancers as well as compared to the normal tissues (Additional file 1: Supplement Fig. 6A and B), which was also reported in a previous study [44, 45], though no significant influence on stomach cancer was found (Additional file 1: Supplement Fig. 6C). Hence, these findings suggested that NQO1 and GSTP1 were upregulated in the tumor tissues of GBM patients, and their expression was correlated.

**Fig. 6:**
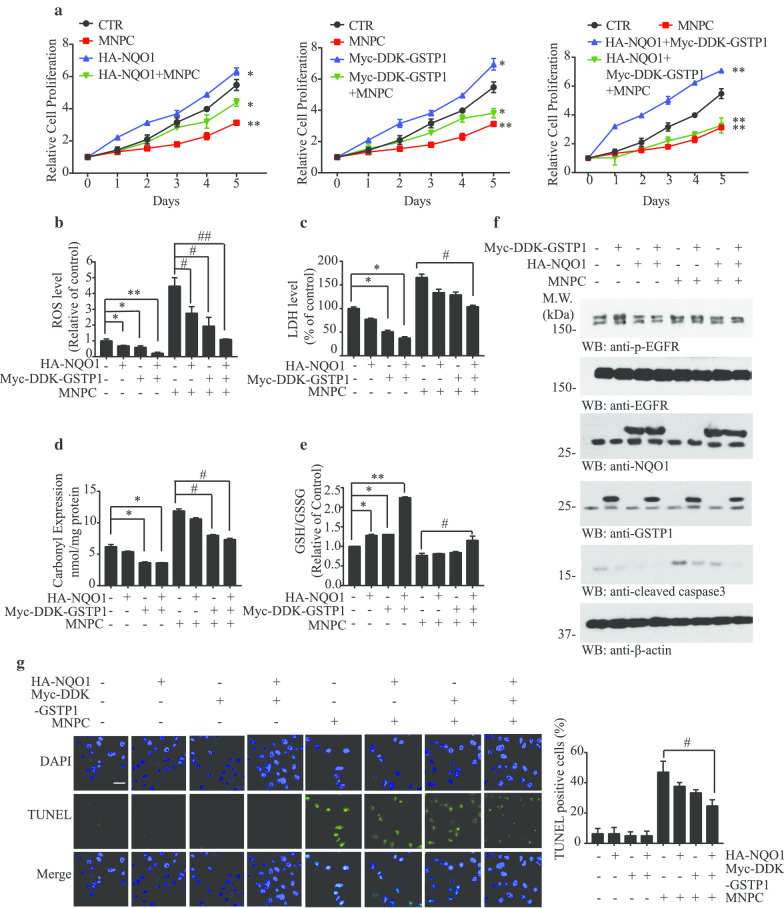
MNPC decreases cell proliferation by overexpressing NQO1 and GSTP1. **a** MTT assay of U87MG/EGFRvIII cells. U87MG/EGFRvIII cells were overexpressed either NQO1 or GSTP1 or both, followed by treatment with MNPC. Cell treated with MNPC (1 μM) or Myc-DDK-GSTP1 or HA-NQO1 or combination for 5 days. **b** The ROS levels, **c** LDH levels, **d** carbonyl expression levels and **e** GSH/GSSG ratios of U87MG/EGFRvIII cells overexpressing NQO1 and GSTP1, followed by MNPC treatment. **f** Western blot analysis of U87MG/EGFRvIII cells treated with MNPC (1 μM) or Myc-DDK-GSTP1 or HA-NQO1 or combination for 48 h. **g** Effect of overexpression on apoptosis confirmed by TUNEL assay. The images of TUNEL-positive cells were captured by a confocal microscope. Scale bar: 20 μm. Data are means ± SEM (*p < 0.05; **p < 0.01; ***p < 0.001, n = 3)

### MNPC antagonizes the oxidative stress reduction mediated by NQO1 and GSTP1

**Fig. 7:**
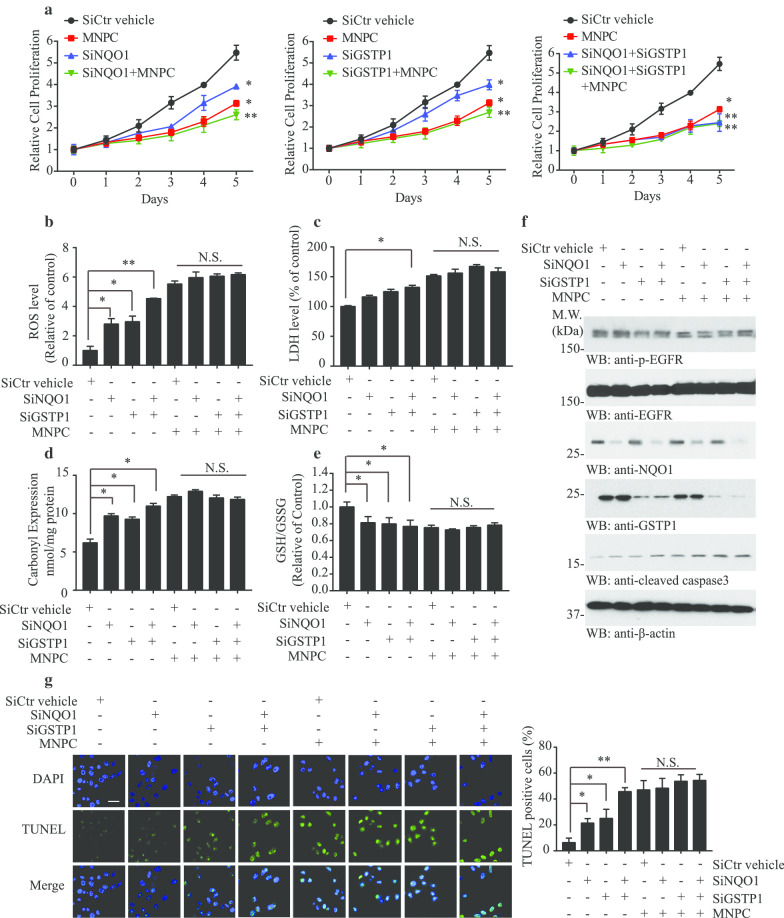
MNPC inhibits cell proliferation by blocking NQO1 and GSTP1 activities. **a** MTT assay of U87MG/EGFRvIII cells. Cell treated with MNPC (1 μM) or si-GSTP1 or si-NQO1 or combination for 5 days. **b** The ROS levels, **c** LDH levels, **d** carbonyl expression levels and **e** GSH/GSSG ratios of depletion of NQO1, GSTP1 and MNPC. **f** Western blot analysis of U87 MG/EGFRvIII cells treated with MNPC (1 μM) or si-GSTP1 or si-NQO1 or combination for 48 h. **g** Effect of siRNA on apoptosis confirmed by TUNEL assay. The images of TUNEL-positive cells were captured by a confocal microscope. Scale bar: 20 μm. Data are means ± SEM (*p < 0.05; **p < 0.01; ***p < 0.001, n = 3)

To further investigate the cell proliferation and ROS status relationship among NQO1, GSTP1 and MNPC, we overexpressed NQO1, GSTP1 or both, treated the cells with MNPC or vehicle in U87MG or U87MG/EGFRvIII cells. As described above, both NQO1 and GSTP1 overexpression enhanced cell proliferation, which was reversed by MNPC (Fig. 6a). Accordingly, overexpression of NQO1 and GSTP1 significantly repressed the ROS levels (Fig. 6b), LDH levels (Fig. 6c) and carbonyl expression (Fig. 6d) in U87MG/EGFRvIII cells but not U87MG cells (Additional file 1: Supplementary Fig. 7A-C), while MNPC antagonized these effects. Moreover, MNPC also reversed the upregulated GSH/GSSH levels induced by NQO1 and GSTP1 (Fig. 6e). Immunoblotting analysis indicated that MNPC increased cleaved-caspase 3 concentrations when both NQO1 and GSTP1 were highly expressed (Fig. 6f), results validated with the TUNEL assay (Fig. 6g). On the other hand, MNPC had no effect on cell number (Fig. 7a), ROS levels (Fig. 7b), LDH level (Fig. 7c), carbonyl expression (Fig. 7d), GSH/GSSH ratios (Fig. 7e) or apoptosis (Fig. 7f and g) in cells in which NQO1, GSTP1 or both were depleted in U87MG/EGFRvIII cells (Fig. 7), supporting the hypothesis that both NQO1 and GSTP1 were the principal cellular targets of MNPC.

### MNPC inhibits the intracranial growth of U87MG/EGFRvIII cells and prolongs mouse survival

**Fig. 8:**
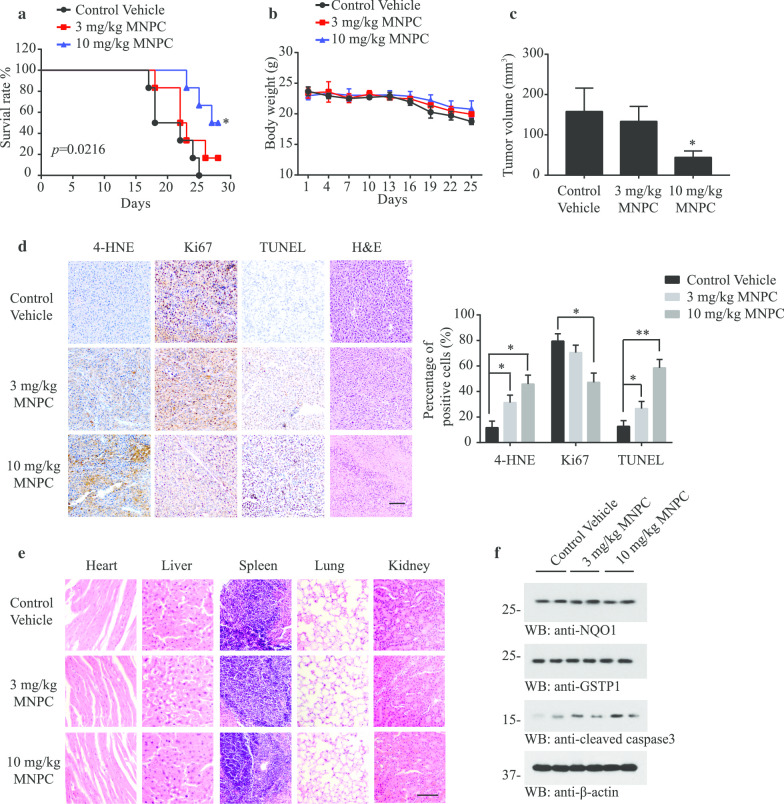
MNPC suppresses tumor growth of U87MG/EGFRvIII cells in the brain and elongates mouse life span. **a** Nu/Nu nude mice were treated with increasing doses (3, 10 mg/kg) of MNPC by i.p. every two days for a total of 10 doses (*n* = 6 mice per group). The survival curves of brain tumor-bearing mice were calculated. **b** Weight of intracranial model mice treated with MNPC (3, 10 mg/kg) or control vehicle. **c** Quantitative analysis of intracranial tumor volume in the mice treated with MNPC (3, 10 mg/kg) or control vehicle. **d** IHC of 4-HNE, Ki67, TUNEL and H&E in tumor nodules from MNPC (3, 10 mg/kg) or control vehicle-treated mice. **e** Chronic MNPC treatment displayed no detectable toxicity. Histological analysis of hematoxylin–eosin-stained tissue sections of representative mice in MNPC or vehicle-treated group. Scale bar: 100 μm. **f** Western blot analysis of intracranial tumor lysates from the animals treated with MNPC (3, 10 mg/kg) or control vehicle. Data are means ± SEM (*p < 0.05, one-way ANOVA)

To assess the therapeutic potential of MNPC, we used a murine intracranial xenograft model [37], inoculated with U87MG/EGFRvIII cells (Additional file 1: Supplementary Fig. 8a). Seven days following tumor injection, the animals were treated with vehicle or different doses of MNPC (3 or 10 mg/kg) every 2 days for a total of 10 doses. MNPC treatment at the higher dose resulted in enhanced mouse survival (Fig. 8a), body weight (Fig. 8b) and reduced tumor growth (Fig. 8c and Additional file 1: Supplementary Fig. 8B). Next, we examined the ROS levels and signaling cascades in the tissue samples collected from tumors extracted from MNPC or vehicle-treated mice. MNPC dose-dependently increased ROS levels and active apoptotic caspase 3 and apoptosis (Fig. 8d and f). H&E staining of major organs demonstrated no detectable toxicity, including kidney, liver, spleen, heart and lung (Fig. 8e), suggesting that MNPC does not have detectable systemic toxicity. We also tested the CBC (complete blood chemistry) from the serum, ALT and AST from the whole blood and found that the side effect of MNPC is modest (Additional file 1: Supplementary Fig. 8C). Together, the data support that MNPC significantly blocks tumor growth by suppressing cell proliferation and inducing apoptosis via escalating ROS in GBM in vivo.

## Discussion

GBM is highly aggressive neoplasia with a dismal prognosis, and patients with EGFR-driven tumors that have absent PTEN do not respond to anti-EGFR therapy [46]. To search for innovative pharmaceutical agents for treating this devastating malignancy, we conducted an uHTS screening and discovered MNPC that selectively blocks the proliferation of U87MG/EGFRvIII as compared to the parental U87MG cells. We have also validated the anti-GBM efficacy with primary GBM cells carrying EGFRvIII mutation versus EGFR WT. To identify its molecular target in EGFRvIII GBM cells that responsible for the preferential anticancer efficacy of MNPC, we employed the affinity column strategy, a frequently used to identify the drug–proteins interactions. Utilizing TIZ-agarose affinity chromatography, we found that both NQO1 and GSTP1 are the major MNPC cellular targets in EGFRvIII GBM cells and identify that MNPC acts as a dual inhibitor, simultaneously blocking both NQO1 and GSTP1. This small molecule tightly binds to the active sites on both enzymes and inhibits their enzymatic activities (Figs. 2 and 3). The precise mechanism of oncogenesis of EGFRvIII is not entirely clear, but involves multiple signaling pathways and the interaction of the mutant EGFRvIII and wt EGFR [47]. Direct inhibition of EGFR signaling in EGFRvIII-expressing cells has not proven to be of great value, due to a number of factors, including cellular plasticity with the compensatory enhancement of other oncogenic mechanisms [48, 49]. Nevertheless, it is worth noting that MNPC does not directly block EGFRvIII; instead, it acts downstream of EGFRvIII signaling via directly blocking both NQO1 and GSTP1 reductases. MNPC may obviate some of these resistance mechanisms by interacting with a particular vulnerability, elevated ROS.

Primary GBMs often possess EGFR amplification, PTEN mutation and loss of chromosome 10, while TP53 mutations are common in secondary GBM, unlike the primary types [50, 51]. These mutations affect the redox balance in the tumor environment. For instance, ligation of EGFR by EGF induces endogenous production of intracellular reactive oxygen species (ROS) and H_2_O_2_ in cancer cell lines [52, 53]. In response to ligation, EGFR forms homo- and heterodimers activating several intracellular signal pathways, such as PI3K/Akt and MAPK, leading to an increase in DNA synthesis [52]. Also, high levels of H_2_O_2_ significantly increase the Tyr autophosphorylation by EGFR, resulting in the generation of ROS [52]. PTEN acts as a tumor suppressor, negatively regulating PI3K/Akt pathway [54, 55]. This phosphatase plays an important role in the regulation of metabolism, apoptosis, cell proliferation and survival, being affected by redox status, specifically by H_2_O_2_, which can oxidize the protein, inducing the formation of a disulfide bond between Cys71 and Cys124 in the N-terminal phosphatase domain [56]. As a result, this leads to alterations in its interaction with signaling and regulatory proteins [56, 57]. Presumably, overexpression and hyperactivation of EGFR might result in an increase in H_2_O_2_ levels, disturbing several signaling pathways and stimulating cell survival and proliferation.

NQO1, originally referred to as DT-diaphorase, is a cytosolic flavoenzyme that plays an important role in protection against endogenous and exogenous quinones by catalyzing two- or four-electron reductions of these substrates [12]. Recently, we reported that NQO1 acts as a downstream target of PTEN in glioblastoma cells, promoting GBM cell proliferation and suppressing ROS [58]. In alignment with its paradoxical roles as both anticancer enzyme and oncogene, NQO1 augments GBM cell growth in response to PTEN expression, which is in sharp contrast to another downstream target of PTEN, PINK1, which also possesses antioxidant activity [59]. Though previous studies demonstrate the importance of NQO1 signaling for the progressive phenotype in colorectal cancer [60] and GSTP1 is overexpressed in many cancers and linked to drug resistance [61], the molecular mechanisms of how these reductive enzymes involved in GBM proliferation remain unclear. Both NQO1 and GSTP1 are well-known phase II metabolism enzymes catalyzing diverse reactions that collectively result in broad protection against electrophiles and oxidants [62]. However, the biological roles of NQO1 and GSTP1 in GBM proliferation are barely known. Interestingly, we show that depletion of NQO1 and GSTP1 strongly inhibits cell growth and induces oxidative stress, especially in U87MG/EGFRvIII cells. Conversely, overexpression NQO1 and GSTP1 promote cancer cell proliferation, supporting that higher NQO1 and GSTP1 levels are essential for cancer cell proliferation and the redox homeostasis (Figs. 6 and 7). Previous studies have indicated that tumors elevate NQO1 to enhance the cell survival and reduction of NQO1 potentially ameliorates the negative effects of tumor-NQO1 overexpression on patient outcome [63]. In addition, the effect of GSTP1 on cell proliferation and apoptosis has been examined in esophageal squamous cell carcinoma cell lines. Knocking down GSTP1 in cancer cells significantly decreases cell proliferation, while early apoptosis occurs [64].

There are two characterized polymorphisms in NQO1 including NQO1*2 (C to T change at position 609 of human cDNA) [65] and NQO1*3 (C465T substitution, resulting in an arginine-to-tryptophan amino acid change in the protein)[66, 67]. NQO1 overexpression increases cell sensitivity to β-lapachone, whereas NQO1*2 polymorphism triggers quinone-based chemotherapies-sensitivity [68]. Both mutations are located far away from the active site of MMPC binding, which theoretically has no significant effects on the inhibitory activity of MNPC. According to the crystal structure, the existence of Pro186 stabilizes the β-sheet. The rigid five-membered ring not only provides hydrophobic interaction but also provides a certain direction for the peptide chain. If it mutates to flexible serine, it may make the β-sheet of protein unstable and cause allosteric regulation, thus reducing the activity of the enzyme. This may be the reason that the enzymatic activity of P186S mutant is very low. For R138W mutation, its position lies in the loop connecting two β-sheets. The mutation may also affect the stability of β-sheet, which also causes some allosteric regulation and reduces the enzyme activity. The mutations caused by these two gene polymorphisms are not in the active center and might not directly affect the inhibition of MNPC. In addition, because MNPC is not a quinone-based NQO1 inhibitor and it does not need NQO1 activation, the reduced enzyme activity of the two mutants will not affect its inhibitory effect. Conceivably, the mutant cells with low NQO1 activity are not dependent on NQO1 and may not be sensitive to NQO1 inhibitors.

GST polymorphisms including Ile105Val polymorphism is involved in the development of glioma and other cancers [69, 70]. The A-G change of GSTP1 Ile105Val polymorphism significantly increased platinum-based chemotherapy response [71]. Our results already showed that this mutation did not affect the inhibitory effects of MNPC, while MNPC had an IC_50_ of 0.40 ± 0.39 μM and 0.65 ± 0.19 μM against wild-type GSTP1 and V104A mutant, respectively (Additional file 1: Supplementary Table 1, Additional file 1: Supplementary Fig. 2F and G). And also K_d_ values of wild-type GSTP1 and V104A mutant were 17.60 ± 0.80 μM and 24.40 ± 1.95 μM, respectively. Hence, NQO1 and GSTP1 polymorphisms with mutations far away from the active site of MMPC binding, so the cells with these polymorphisms might be still sensitive to MNPC inhibition.

Nitazoxanide (NTZ) is a broad-spectrum antiparasitic and broad-spectrum FDA-approved drug for cryptosporidium infection [72]. Chemically, nitazoxanide is the prototype member of the thiazolides, a class of drugs which are synthetic nitrothiazolyl–salicylamide derivatives with antiparasitic and antiviral activity [73]. Tizoxanide, an active metabolite of nitazoxanide in humans, is also an antiparasitic drug of the thiazolide class [74]. In our study, tizoxanide displays similar structure–activity relationship with MNPC, which suggests that TIZ, NTZ and MNPC may share the same targets in GBM. NTZ also depolarizes the mitochondrial membrane along with the inhibition of NQO1 [75], which further supports our findings. Taken together, our findings strongly support that NTZ and TIZ may be repurposed for treating the devastating GBM. The relative efficacy of MNPC as compared to NTZ and TIZ in vivo remains to be determined.

## Conclusions

In our study, we show that MNPC simultaneously inhibits both NQO1 and GSTP1 enzyme activities via binding to both enzymes’ active sites (Fig. 2 and Additional file 1: Supplementary Fig. 2). It is not unusual for a small molecule that acts as a dual inhibitor. For instance, a previous study demonstrated that dual BACE-1/GSK-3β inhibitors act through the inhibition of the NQO1 enzyme in order to counteract the oxidative stress in Alzheimer's disease [76]. Dual GSTP1 and HIF1α inhibitor induce autophagy and apoptosis in HepG-2 cells [77]. Furthermore, we show that the cytotoxic and anti-proliferative effects of MNPC are due to inducing apoptosis by activating caspase 3, which is a key member of the caspase signaling pathway and the most important executor of cell apoptosis. Notably, caspase 3 can be activated by oxidative stress [78]. Therefore, it is not surprising that inhibition of NQO1 and GSTP1 by MNPC results in oxidative stress escalation in U87MG/EGFRvIII cells, leading to cell apoptosis (Fig. 3i). Possibly because MNPC targets a specific vulnerability in EGFRvIII cells, MNPC treatment appeared to be relatively nontoxic in mice, with an absence of weight loss or any overt damage of major organs. The virtual ADMET analysis reveals that MNPC possesses acceptable ADMET profiles [79, 80]. In conclusion, targeting GSTP1 and NQO1 in general, and MNPC, specifically, are potentially promising therapeutic strategies for treating EGFRvIII-expressing GBM without demonstrable toxicities by taking advantage of a selective vulnerability that develops because of the generation of high levels of ROS species in these cells. GSTP1 and NQO1 are then needed to detoxify these ROS species and MNPC, through inhibition of both enzymes attacks this “Achilles heel.” Conceivably, the development of MNPC as a novel anticancer agent will provide an unprecedented strategy for GBM cancer therapy.

## Supplementary information

**Additional file 1**:** Figure S1**, related to Fig. 1, presents SAR analysis of the anti-proliferative effects of the MNPC derivatives.** Figure S2**, related to Fig. 2, presents MNPC dose-dependently blocks primary GBM sphere formation capacity and FDA-approved NTZ and TIZ exhibit selective inhibition against U87MG/EGFRvIII cells.** Figure S3**, related to Fig. 3, presents MNPC displays no inhibition against cell proliferation in normal cells and invasion or AEP enzyme in U87MG EGFRvIIII cells.** Figure S4**, related to Fig. 4, additionally shows that NQO1 and GSTP1 selectively mediate U87MG/EGFRvIII but not U87MG cell proliferation and oxidative stress.** Figures S5** and** Fig. S6** show that NQO1 and GSTP1 are highly positively correlated in GBM.** Figure S7**, related to Figs. 6 and 7, presents NQO1 and GSTP1 overexpression in U87MG cells and does not affect the parental U87MG proliferation.** Figure S8**, related to Fig. 8, presents MNPC suppresses tumor growth of U87MG/EGFRvIII cells and elongates the nude mouse life span.** Table S1** provides information on binding affinities and inhibitory effects of MNPC with GSTP1 and its mutants.** Table S2** presents crystallographic data and refinement statistics.** Table S3** provides the primers used for mutagenesis. (PDF 3953 kb)

## Data Availability

The datasets generated and analyzed during the current study are not publicly available due to the ongoing study but are available from the corresponding author on reasonable request.

## Abbreviations

GBM: Glioblastoma
EGFR: Epidermal growth factor receptor
NQO1: NADPH quinone oxidoreductase 1
GSTP1: Glutathione-S-transferase Pi 1
ROS: Reactive oxygen species
RTK: Receptor tyrosine kinase
GSTs: Glutathione S-transferases
HTS: High-throughput chemical screening
SAR: Structure–activity relationship
MNPC: 5-Methyl-N-(5-nitro-thiazol-2-yl)-3-phenylisoxazole -4-carboxamide
NTZ: Nitazoxanide
TIZ: Tizoxanide
