# Supplementary material for: Discovery of a dual inhibitor of NQO1 and GSTP1 for treating glioblastoma
Source: J Hematol Oncol. 2020 Oct 21;13:141. doi: 10.1186/s13045-020-00979-y (PMC7579906; doi:10.1186/s13045-020-00979-y)
Supplement: Supplementary file 1 — Additional file 1: Figure S1, related to Fig. 1, presents SAR analysis of the anti-proliferative effects of the MNPC derivatives. Figure S2, related to Fig. 2, presents MNPC dose-dependently blocks primary GBM sphere formation capacity and FDA-approved NTZ and TIZ exhibit selective inhibition against U87MG/EGFRvIII cells. Figure S3, related to Fig. 3, presents MNPC displays no inhibition against cell proliferation in normal cells and invasion or AEP enzyme in U87MG EGFRvIIII cells. Figure S4, related to Fig. 4, additionally shows that NQO1 and GSTP1 selectively mediate U87MG/EGFRvIII but not U87MG cell proliferation and oxidative stress. Figures S5 and Fig. S6 show that NQO1 and GSTP1 are highly positively correlated in GBM. Figure S7, related to Figs. 6 and 7, presents NQO1 and GSTP1 overexpression in U87MG cells and does not affect the parental U87MG proliferation. Figure S8, related to Fig. 8, presents MNPC suppresses tumor growth of U87MG/EGFRvIII cells and elongates the nude mouse life span. Table S1 provides information on binding affinities and inhibitory effects of MNPC with GSTP1 and its mutants. Table S2 presents crystallographic data and refinement statistics. Table S3 provides the primers used for mutagenesis. (PDF 3953 kb) [file 13045_2020_979_MOESM1_ESM.pdf]

Supplementary Figure 1

A

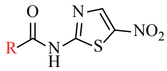

Compound	Structure	IC50（μM）U87MG/ EGFRvIII	IC50 (μM) U87MG
20	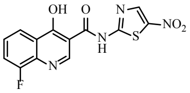	1.42	18.94
13	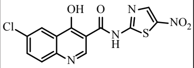	1.55	13.32
11	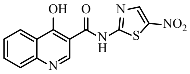	1.89	17.11
12	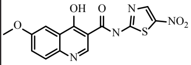	2.37	21.14
14	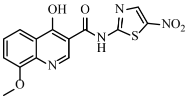	2.61	19.83

B

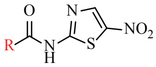

Compound	Structure	IC50(μM) U87MG/ EGFRvIII	IC50 (μM) U87MG
19	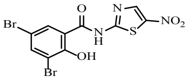	3.03	35.41
1	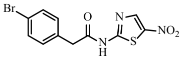	3.59	33.12
2	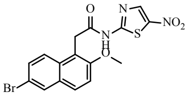	4.81	36.81
16	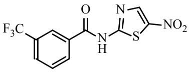	4.83	25.12
18	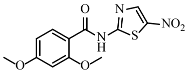	5.03	39.16

C

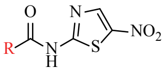

Compound	Structure	IC50(μM) U87MG/ EGFRvIII	IC50 (μM) U87MG
7	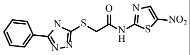	4.81	25.11
8	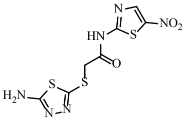	5.35	19.98
6	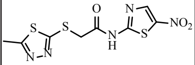	5.79	23.12
10	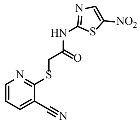	7.14	26.81
9	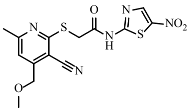	7.23	25.17

D

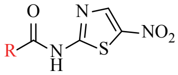

Compound	Structure	IC50(μM) U87MG/ EGFRvIII	IC50 (μM) U87MG
17	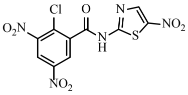	3.61	15.33
15	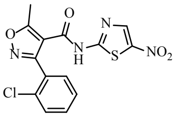	6.22	27.64
3	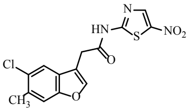	6.98	31.74
4	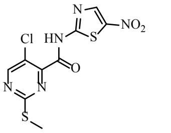	7.18	25.31
5	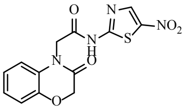	8.11	31.15

Supplementary Figure 2

A

HK296 – EGFRvIII

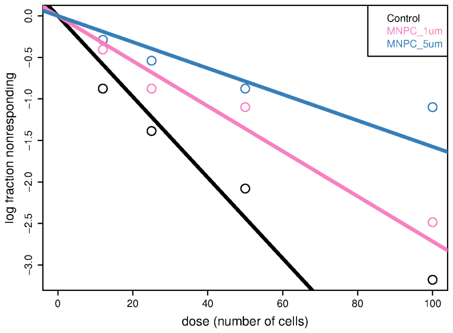

HK296 – EGFRvIII

Group	Stem cell frequency	*p*-value vs control
Control	1/21.1	-
MNPC_1μM	1/37.3	0.0045
MNPC_5μM	1/64	6.17 E-08

B

HK408 – no EGFRvIII

Control MNPC_1μM MNPC_5μM

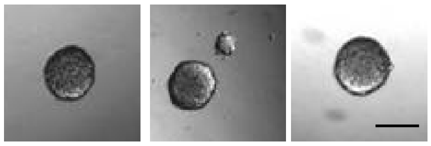

HK408 – no EGFRvIII

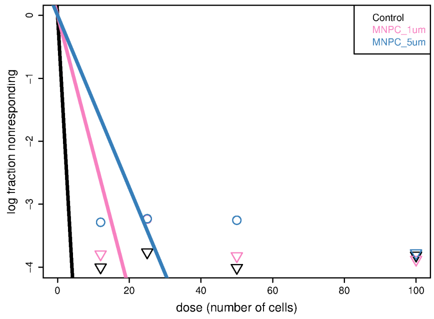

C

HK157 – no EGFRvIII

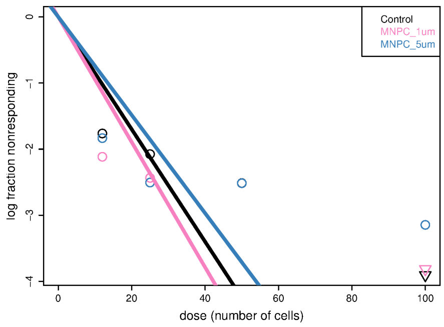

D

NTZ

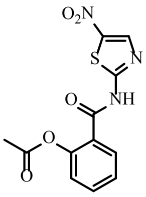

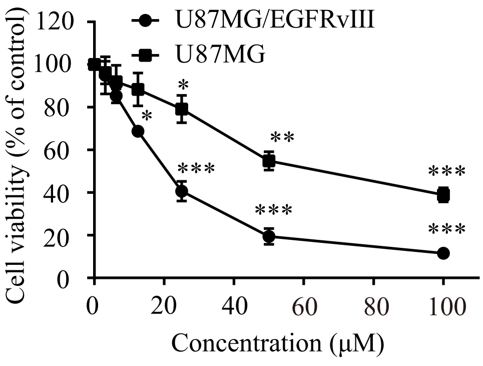

U87MG IC50: 66.7 μM

U87MG/EGFRvIII IC50: 19.4 μM

E

TIZ

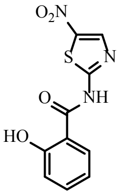

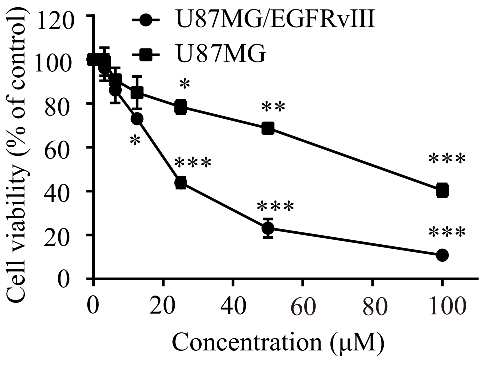

U87MG IC50: 82.9 μM

U87MG/EGFRvIII IC50: 22.4 μM

F

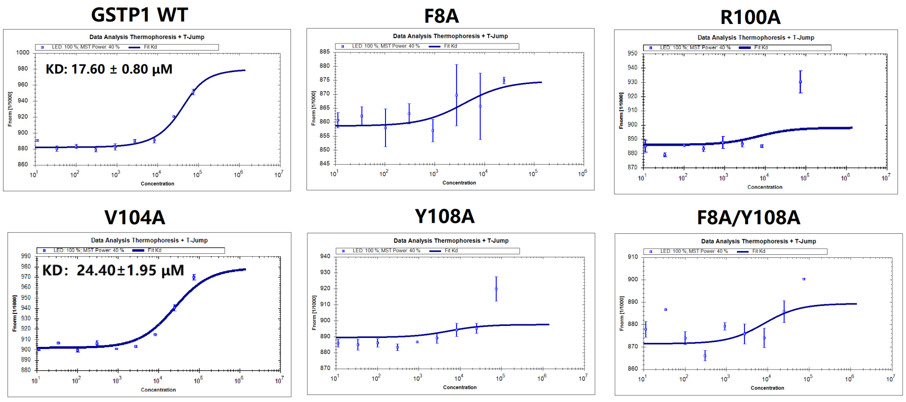

G

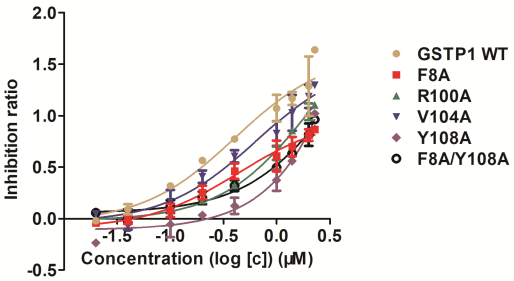

Supplementary Figure 3

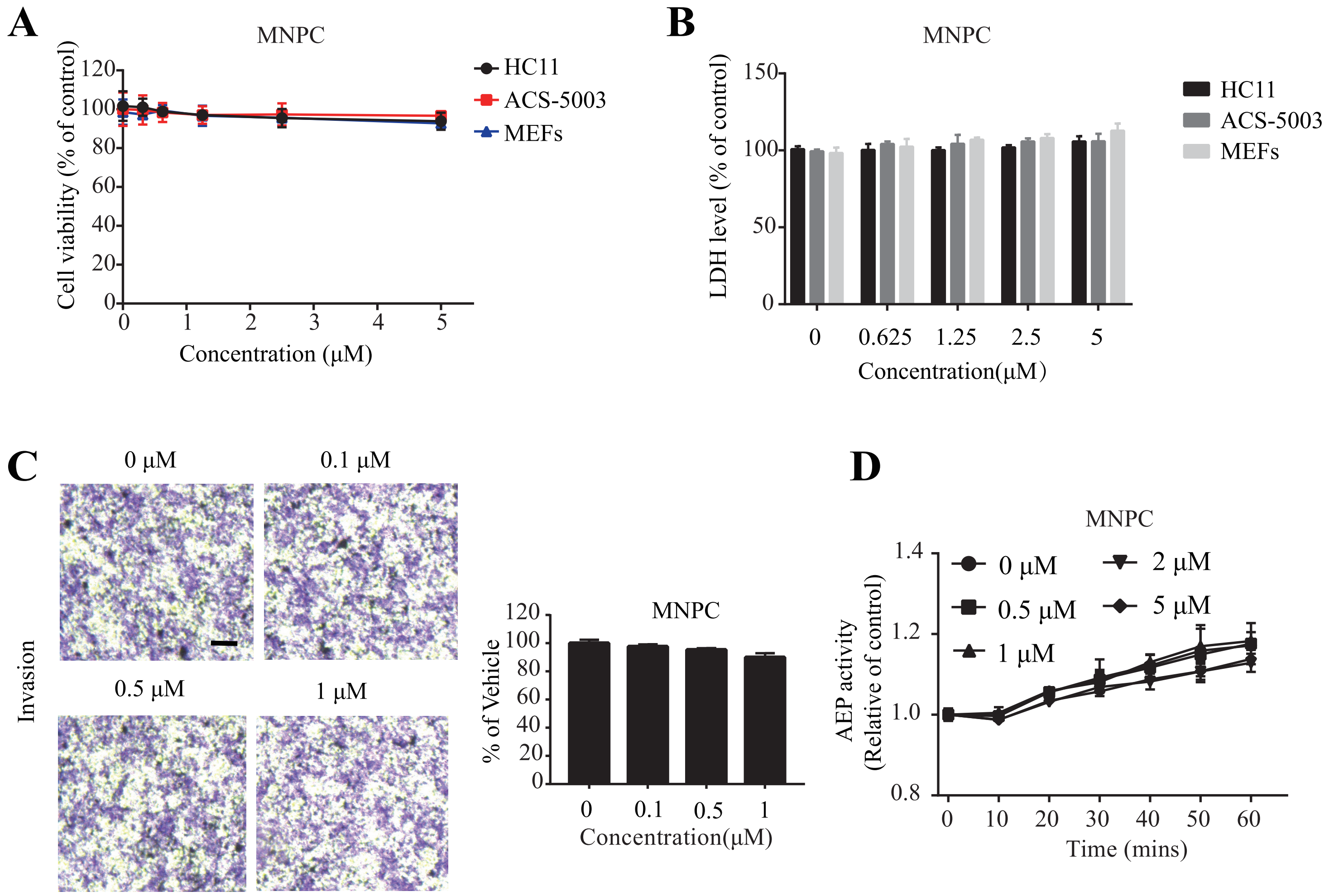

Supplementary Figure 4

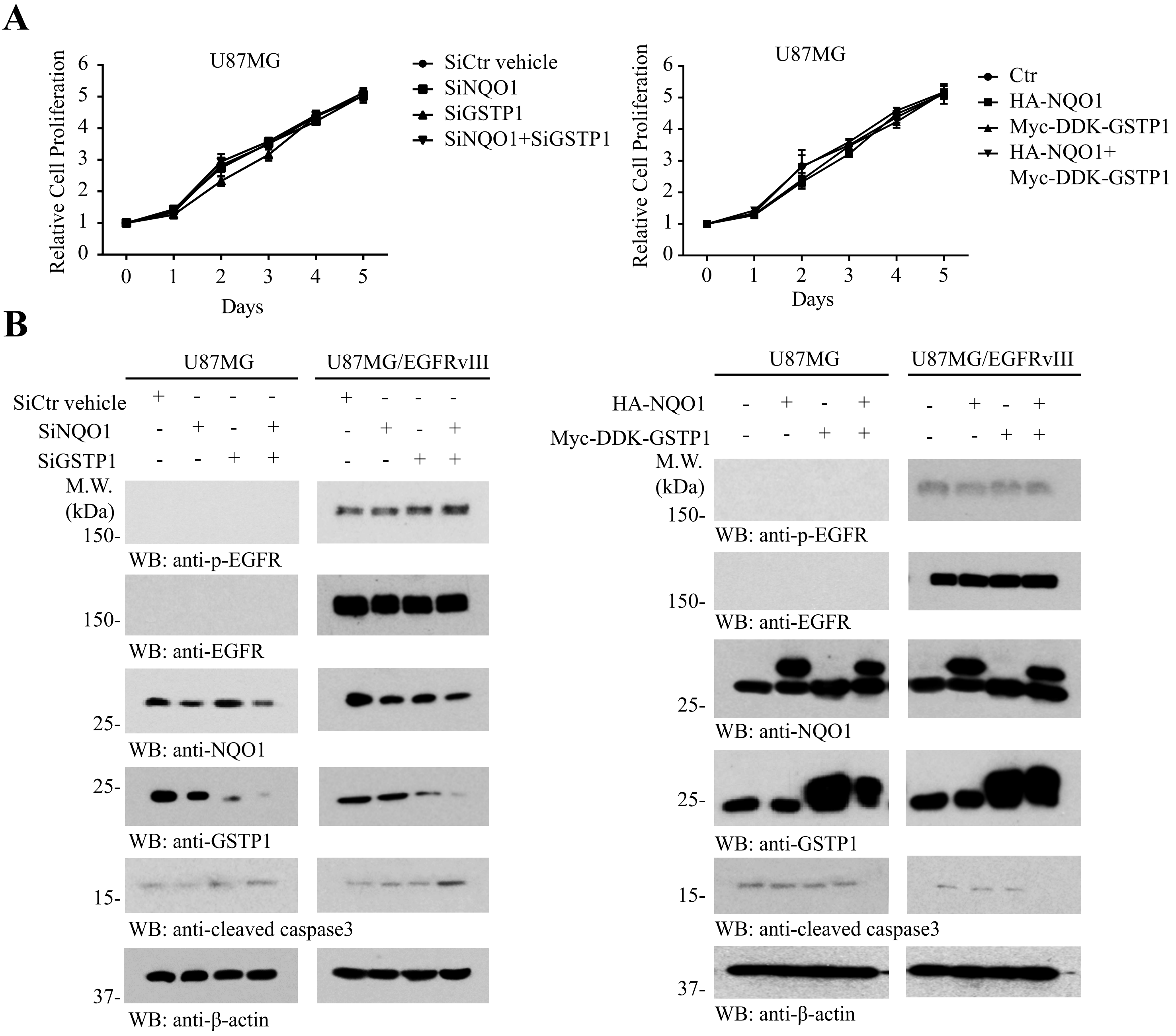

Supplementary Figure 5

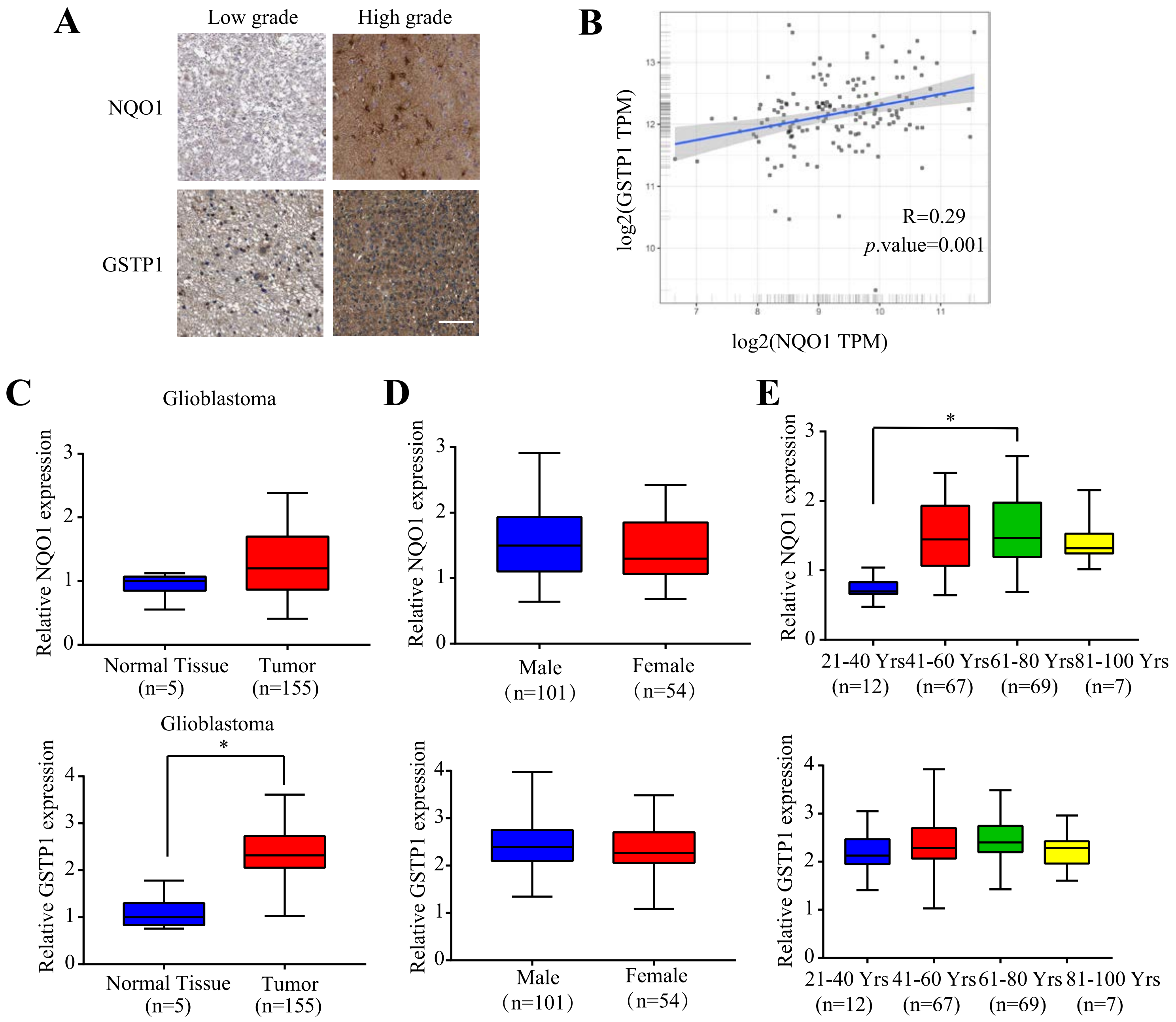

Supplementary Figure 6

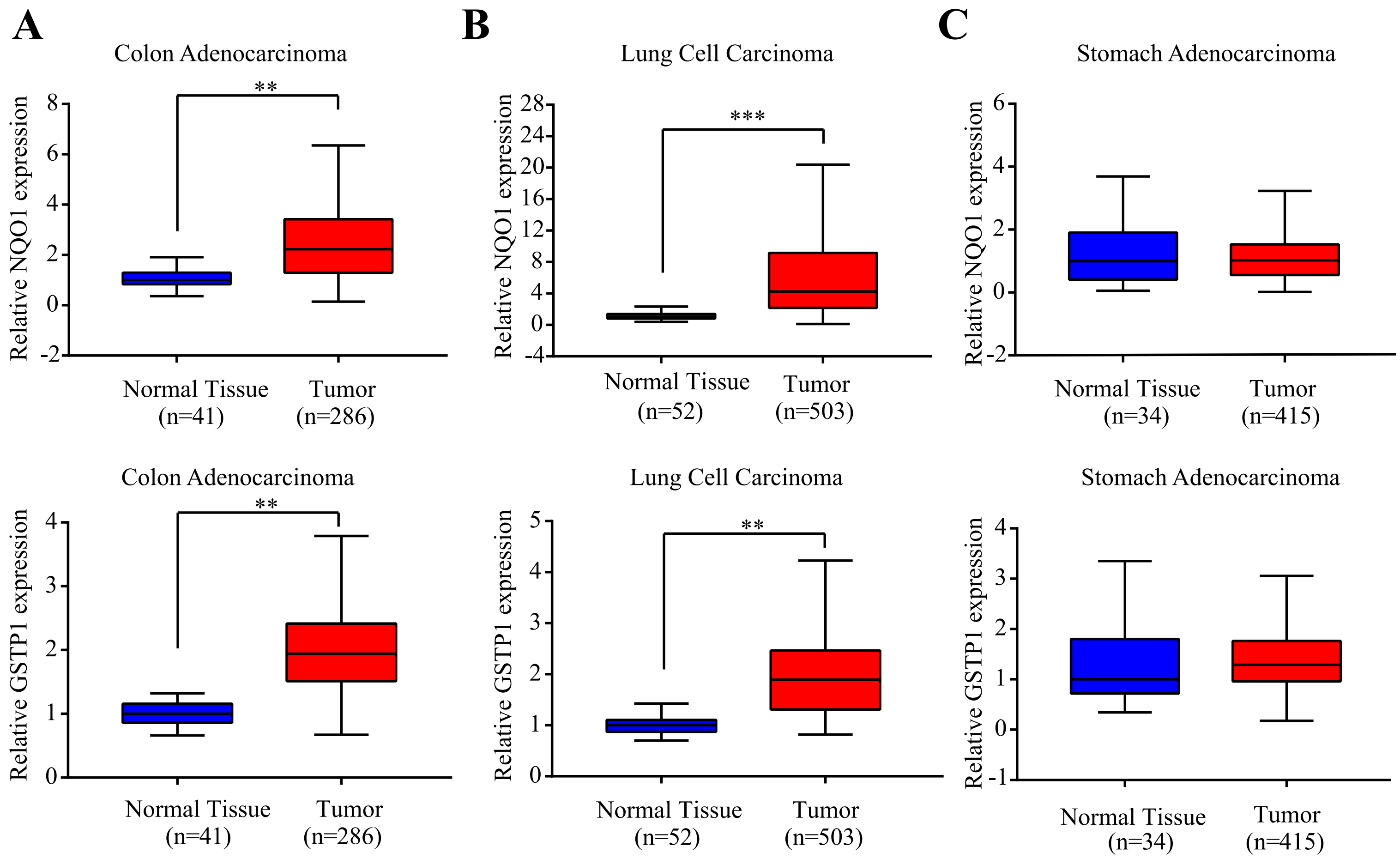

Supplementary Figure 7

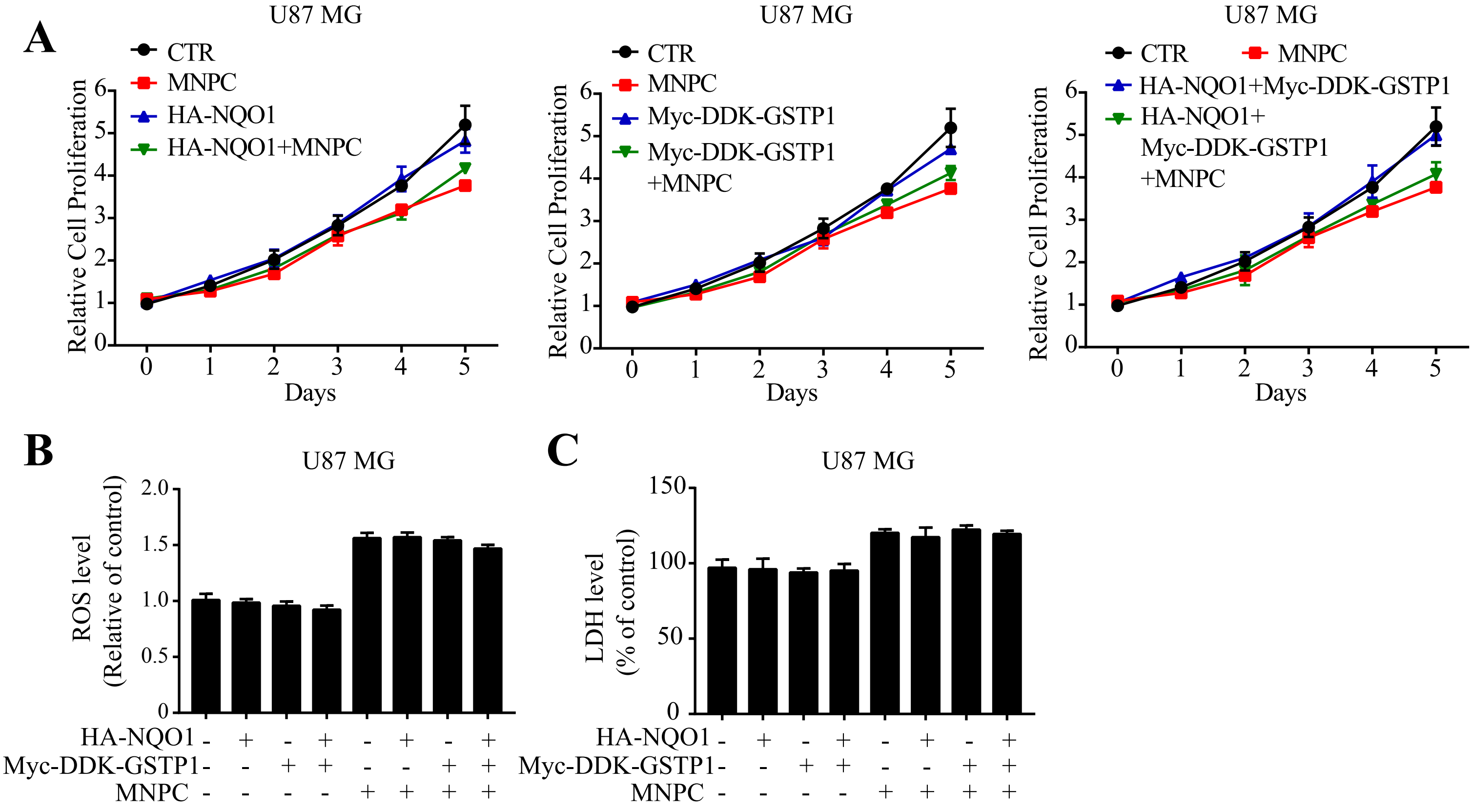

Supplementary Figure 8

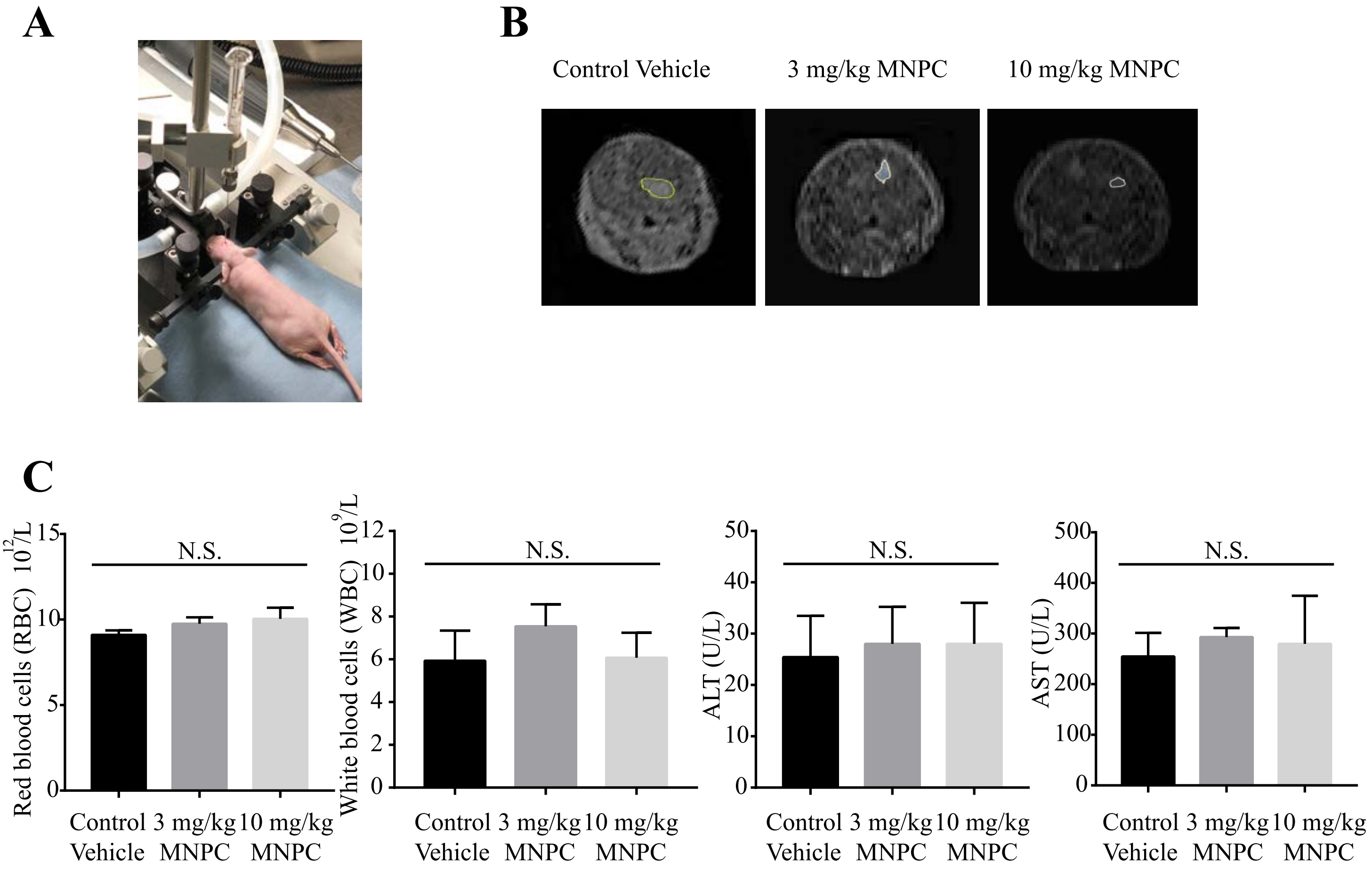

Supplementary Table 1. Binding affinities and inhibitory effects of MNPC with GSTP1 and its mutants.

Protein	Kd (μM) [a]	IC_{50} (μM)
GSTP1 WT	17.60 ± 0.80	0.40 ± 0.39
F8A	n.b.[b]	2.42 ± 0.36
R100A	n.b.	2.37 ± 0.38
V104A	24.40 ± 1.95	0.65 ± 0.19
Y108A	n.b.	25.74 ± 1.41
F8A/Y108A	n.b.	4.01 ± 0.60

Each value was expressed as mean ± SEM(n=3).

[a]The Kd value is automatic calculated by the curve fitting, and presents as means ± SEM for three experiments.

[b]n.b. is no clear binding detected in the MST measurement.

Supplementary Table 2. Crystallographic Data and Refinement Statistics.

Parameters	NQO1/MNPC complex	GSTP1
**Data collection**		
Space group	P41	P212121
Cell dimensions		
a, b, c (Å)	47.882, 147.882, 209.791	69.655,82.179， 89.324
α, β, γ (°)	90, 90, 90	90, 90, 90
Resolution range (Å)	32.66-2.50(2.54-2.50)	24.11-1.58(1.62-1.58)
Rsym (%)	9.6 (60.5)	9.4 (23.8)
Completeness (%)[a]	99.8 (99.8)	99.7 (99.4)
Redundancy	6.8 (6.0)	5.4 (5.2)
$I/\sigma I$[a]	13.9 (1.8)	32.6 (10.9)
**Refinement**		
Total reflections	154628	70461
R_{work}/R_{free}[c]	0.222,0.264	0.200, 0.223
No. atoms		
Protein	25956	3378
Ligand	853	33
Water	249	426
Average *B*-factor (Å2)	36.3	11.0
Ramachandran Plot (%)		
Favored	94.76	97.14
Allowed	5.14	2.62
Outliers	0.1	0.24
Root mean square deviations		
R.m.s.d. bond lengths (Å)	0.008	0.005
R.m.s.d. bond angle (°)	0.946	0.875

Values in parenthesis are for highest resolution shell. 5 % of the data was used in the Rfree calculation.

Supplementary Table 3. The primers used for mutagenesis.

	Nucleotide sequence
F8A forward	AAGCCAGTATACACTCCGCTATC
F8A reverse	CGAACTGGGGCATAGACCACGGTGTAGGG
F8A forward1	TGGTCTATGCCCCAGTTCGAGGCCGCTGC
F8A reverse1	GATAGCGGAGTGTATACTGGCTT
R100Aforward	AAGCCAGTATACACTCCGCTATC
R100A reverse	AGATGAGGGAGATGTATTTGCAGGCGAGGTCCT
R100A forward1	CTGCAAATACATCTCCCTCATCTACACCAACTA
R100A reverse1	GATAGCGGAGTGTATACTGGCTT
V104A forward	AAGCCAGTATACACTCCGCTATC
V104A reverse	ATGAGGGAGGCGTATTTGCAGCGGAGGTC
V104A forward1	GCAAATACGCCTCCCTCATCTACACCAAC
V104A reverse1	GATAGCGGAGTGTATACTGGCTT
Y108A forward	AAGCCAGTATACACTCCGCTATC
Y108A reverse	CGATGAGGGAGATGTATTTGCAGCGGAGGTCCT
Y108A forward1	CTGCAAATACATCTCCCTCATCGCCACCAACTA
Y108A reverse1	GATAGCGGAGTGTATACTGGCTT
F8A/Y108A forward	AAGCCAGTATACACTCCGCTATC
F8A/Y108A reverse	CGATGAGGGAGATGTATTTGCAGCGGAGGTCCT
F8A/Y108A forward1	CTGCAAATACATCTCCCTCATCGCCACCAACTA
F8A/Y108A reverse1	GATAGCGGAGTGTATACTGGCTT
